# Vowel alternation with final *i* offers an easy-to-learn morphological option for a sex-blind grammatical gender in French

**DOI:** 10.3389/fpsyg.2024.1310475

**Published:** 2024-02-22

**Authors:** Marie-Claude Marsolier, Pris Touraille, Marc Allassonnière-Tang

**Affiliations:** ^1^CEA, CNRS, Institute for Integrative Biology of the Cell (I2BC), Université Paris-Saclay, Gif-sur-Yvette, France; ^2^CNRS, UMR 7206 Éco-anthropologie, Muséum National d’Histoire Naturelle, Université Paris Cité, Paris, France

**Keywords:** sex-based grammatical gender, sex-blind grammatical gender, French, gender-neutral language, language change, non-binary identities, epistemological break tool, vowel alternation

## Abstract

Like all modern Romance languages, French has a sex-based grammatical gender with two genders, feminine and masculine, and a lexicon that is highly sex-differentiated. These characteristics give rise to a number of issues, including the problematic generic use of the masculine grammatical gender, coupled with the challenge of sex categorization itself, and the epistemological difficulty of an adequate sociological description and analysis of what gender commonsense categories really are about. To remedy these concerns, several authors have proposed the creation of an additional, epicene grammatical gender. We have identified three such systematic proposals, or solutions, which specify various morphological options for new epicene nouns and gender markers on their satellite elements. These options include the use of non-standard or rarely used characters, the merging of feminine and masculine gender markers, as well as consonantal and vowel changes. In the simplest proposal, referred to as “solution I,” new epicene forms are mostly derived from feminine forms by systematically replacing with an *i* the final *e* that generally differentiates feminines from their masculine counterparts in written French. Although these solutions are used in some communities, their learnability has not been addressed so far, even though it could be a determining factor in their popularity and their eventual integration into standard French. In the present study, we provide a first assessment of this aspect by means of an online translation test. For each solution, French-speaking participants were instructed that they would be trained to learn an “alien” language that does not mark sex/gender categories (these alien languages correspond to standard French where only gendered words referring to people are replaced by the new epicene forms recommended by each solution). After a short learning-by-example phase, participants were required to translate into the alien language a set of 16 standard French sentences. The translations were analyzed as a function of several variables including the participants’ self-reported age and sex, the word categories and the solutions themselves. While all solutions proved quickly learnable, participants’ responses with solution I achieved the highest accuracy score, in particular with regard to the production of non-standard epicene forms.

## 1 Introduction

Like around 20% of the world’s languages (Allassonnière-Tang et al., 2021), French has grammatical gender (i.e., a noun classification system) whose semantic basis is one of the commonsense “sex” categorization. Every noun in standard French belongs to either the feminine or the masculine gender. This gender categorization controls the morphological variations of the satellite elements, also called “agreement targets,” which determine a noun or refer to it (determiners, adjectives, pronouns). In terms of grammatical gender assignment, for people and other familiar animals, grammatical gender reflects the female/male categories people are culturally supposed (and obliged) to classify themselves into, while nouns referring to objects are either feminine or masculine. There are only a few exceptions to this rule, including feminine or masculine nouns interpreted with a generic value, such as *un individu* (‘an individual’) or *une personne* (‘a person’). Apart from these few words, grammatical gender functions in French as a systematic “sex categorization device.” This grammatical marking is combined with a lexical marking, since a given social role is generally denoted by two nouns, one feminine, the other masculine, whose forms are often different, e.g., *musicienne*_fem_ ‘female musician’ vs. *musicien*_masc_ ‘male musician’, *mère* ‘mother’ vs. *père* ‘father’ (to facilitate reading of the examples, feminine and masculine related forms are differentiated here with “fem” and “masc” in subscript).

### 1.1 Two divergent critical standpoints for existing propositions of linguistic change

Sex categorization in language gives rise to (at least) two unescapable epistemological critiques that actually lack to be clearly distinguished in the French academic context. We try to present them in some detail in this introduction for they illuminate why the strategies of language change they brought should be recognized as conceptually incompatible (this being valid for all Romance languages).

#### 1.1.1 The “inclusive” solution: an answer to the masculine generic value

The first major critique was (and still is) about the masculine “generic value” (Elmiger, 2013), discussed for half a century now in feminist academic literature as a false generic and as a particularly crafty device in the service of the male hegemonic standpoint (Livia, 2001). The feminist epistemological and political response in French-speaking countries has been, for more than 30 years now, the now labeled “non-sexist,” “inclusive” language (Labrosse, 1996; Viennot et al., 2018), which proposes (among other strategies) new, “contracted double forms” combining feminine and masculine gender markers (e. g. *étudiant.e* corresponding to the full double form *étudiante*_fem_ *ou étudiant*_masc_ ‘female or male student’). The “inclusive” neo-formalisms have met with now legendary oppositions in France, held by language institutions (Académie française, 2017), scholars in linguistics (Szlamowicz, 2018; Grinshpun and Szlamowicz, 2021), and political instances. For example, some members of the French Parliament have recently proposed to officially forbid language amendments and to impose “masculine generics” by law (Chudeau and Auzanot, 2023). In the meantime, a growing corpus of psycholinguistic studies has confirmed the sexist bias identified by feminist epistemologies and demonstrated that “masculine generics” induce a strong male bias in mental representations (Brauer and Landry, 2008; Gygax et al., 2008), with significant consequences on the perception of women’s social (including professional) positions (Chatard et al., 2005; Sczesny et al., 2016). In response to this robust scientific argument, with feminist linguist scholars arguing repetitively against the defense of linguistic *statu quo* (Candea and Véron, 2021), “inclusive language” has now been adopted in numerous academic and social policy spheres in the Francophonie. It actually comprises hybrid and non-stabilized strategies: use of double or contracted feminine and masculine forms, on the one side, and use of native epicene words whenever they can substitute for “masculine generics,” on the other side. But this very solution has an epistemic side effect, particularly highlighted by the categorical “doubling” procedure: the female/male duality appears more philosophically and culturally legitimate than ever. This brings us to the second major philosophical and epistemological critique of sex categorization in language, largely and sometimes voluntarily unrecognized by the current proponents of “inclusive language”: sex categorization as a problem in itself.

#### 1.1.2 The “non-binary” solution: an answer to sex categorization itself

“Sexual difference” is an old axiom of Western philosophical thought, which came to be largely and newly justified with the rise of modern biological sciences (Laqueur, 1992). Moreover, psychoanalysis theory, especially in its French Lacanian heritage, maintains the cultural necessity of sex differentiation and constitutes a strong philosophical standpoint in France. The recognition of the female/male duality as a basis of “humanness” has been reaffirmed recently in the name of an egalitarian, heterosexual, and laic feminism (Agacinski, 2001). Despite this conservative context, sex categories in language have been strongly attacked by French novelist and lesbian theorist Wittig (1992), and designated as the major linguistic device that modern Western societies use to hide the political nature of a renewed heterosexual social order. The international success of Berkeley University philosopher and lesbian theorist Judith Butler contributed, from the 1990’s on, to spread the silenced wittigian critical standpoint (Butler, 1990). The radically new cultural understanding that butlerian analysis offered on hegemonic sexuality (followed by institutionalization of Sexuality and Queer studies) supports a still ongoing epistemic revolution: a growing number of people worldwide, under the LGBTIQA+ banner, choose today to get rid of the obligatory female/male identifications, whether in appearance or language, creating the need of a radically de-sexed language (which started with the use of alternative gender-blind pronouns in English (Baron, 2020), a practice now generalized in social science international conference panels). The non-binary proposal is, to a certain point, sustained, but also instigated, by the fact that female/male categorization is now denied of heuristic value by some authors for many fields of biology (Touraille, 2016) and medical sciences (Lemarchand, 2023). The philosophical need to create a “non-binary grammar” (Coutant et al., 2015) and “non-gendered,” “neutral,” “post-binary” or “epicene” modes of expression coherently blind to sex categorization is progressively emerging in French-speaking countries (Borde, 2016; Martin, 2016; Alpheratz, 2018; Ashley, 2019; Swamy and Mackenzie, 2022). Because they also solve the “masculine generics” epistemic problem, some non-binary grammatical projects have also been labeled “inclusive” by their proponents (Alpheratz, 2018). These terminological choices create a confusion with the “inclusive” original label (which aims toward a “women” category more visible in language), making it difficult to acknowledge the radical epistemic difference existing between language change alternatives.

#### 1.1.3 Toward a solution that would answer both critical standpoints?

Apparent terminological ecumenism, which is clearly a form of political strategy, presently leads to a new epistemic problem aptly named “gender trinarism” (coexistence of non-binary, masculine and feminine “gender identities”). Since in this case the concept of non-binary category applies exclusively to non-binary people and cannot function as a generic, “gender trinarism” is denounced as opposing the “gender decategorization” project (Katz, 2007). Accordingly, an epicene grammatical gender should not be considered as a support for social “identities” (be they “essentialists” or politically constituted), but as an “empty category” (Matushansky, 1998) encompassing everyone. This last critique opens up on the general need of this category for science and for social policy at large. Indeed linguistic female/male classification is now recognized by some French jurists as a problem in judicial documents (Catto, 2023), and, similarly, it has been recently argued that linguistic female/male classification constitutes for scientific thought in general, and for gender studies in particular, what French epistemologist Bachelard (2002) named an “epistemological obstacle.” The ongoing use of sex-based grammatical gender by sociological literature, which endorses the commonsense naturalized gender categories that sociology and gender studies seek to deconstruct, does not permit a rigorous analysis of the gender device in Western societies (Touraille and Allassonnière-Tang, 2023). An epicene grammatical gender would thus serve as a suspensory *tool* for this necessary “epistemological break” in social sciences. It should be noted that this well-identified scientific need conflicts with an epicene grammatical gender that would societally correspond to a new “identity” marker. The three coherent epicene solutions that currently exist for the French language—which our article will formally compare —do not in fact conceptually entail any “gender trinarism.” They merely attempt to systematically get rid of masculine and feminine forms when it comes to people.

### 1.2 The three solutions existing in French for a new epicene grammatical gender

Even if the marking of the female/male categories can be reduced by the use of already existing French epicene words or expressions (e.g., *élève* ‘student’ instead of *étudiant*_masc_ ‘male student’, *le public* ‘the audience’ instead of *les spectatrices*_fem_ *et les spectateurs*_masc_ ‘female and male spectators’, *la personne responsable de l’électricité* ‘the person in charge of the electricity’ instead of *l’électricien*_masc_ ‘the male electrician’), this approach cannot provide a completely epicene mode of expression since not all nouns have an epicene counterpart and not all adjectives and determiners have an epicene form. Furthermore, changing nouns to their definitions (cf. the example of the electrician above) significantly lengthens the content of speech and text. The only effective solution for French, as for other languages with a sex-based grammatical gender, and with a lexicon that is largely sex-differentiated, consists in two parts: (i) extending the lexicon by proposing additional epicene nouns in cases where the corresponding feminine and masculine nouns differ (e.g., *autrice*_fem_/*auteur*_masc_ ‘female/male writer’), and (ii) creating a new grammatical gender, specific to epicene nouns denoting persons, and characterized by new agreement markers, different from those of the feminine and the masculine. Obviously, the new epicene forms only apply to nouns designating persons and their satellite elements, but not to nouns designating objects or concepts and their related elements.

Two authors, Borde (2016) and Alpheratz (2018), have recently theorized the need for a new grammatical gender of that kind in French, that they have called *universel* ‘universal’ or *universaliste* ‘universalist’, and *neutre* ‘neutral’, respectively, and they have put forward concrete, detailed proposals for its new forms. Below we summarize the main features of these proposals, focusing on the procedures for producing new epicene nouns and inflected forms of adjectives. For the sake of clarity, only singular forms are mentioned here: in the cases we cover, the plurals of epicene words are almost always produced by adding a final *-s* (the main exception to this rule concerns the epicene marker *x* in Alpheratz’s proposal, as indicated below). A more detailed description of these solutions is provided in the Supplementary Table S1.

Borde (2016) proposes different procedures to produce new epicene nouns and markers on agreement targets (clearly these proposals only concern forms that are not epicene in standard French). For homophonous feminine and masculine forms that do not end in *-elle*_fem_/-*el*_masc_, Borde suggests an epicene form marked by the unpronounced character *ë* that distinguishes itself from the feminine and masculine forms in writing but not in speech. If we consider the feminine/masculine word pairs *admirée*_fem_/*admiré*_masc_ ‘admired’, *amie*_fem_/*ami*_masc_ ‘female/male friend’, *nue*_fem_/*nu*_masc_ ‘nude’, *docteure*_fem_/*docteur*_masc_ ‘female/male doctor’ for example, the corresponding epicene forms are *admiréë*, *amië*, *nuë*, and *docteurë*, respectively. For homophonous feminine and masculine forms ending in *-elle*_fem_/-*el*_masc_, for example *intellectuelle*_fem_/*intellectuel*_masc_ ‘female/male intellectual’, Borde suggests an homophonous epicene form ending in *-èle*: *intellectuèle*. When the feminine and masculine forms of a word pair are not homophonous, Borde suggests to produce the corresponding epicene form by merging the masculine and feminine endings, in that order. Borde distinguishes three cases: the first case corresponds to a simple merging of the masculine and feminine endings, which gives, for example, *nouveaulle* (for *nouvelle*_fem_/*nouveau*_masc_ ‘new’), *agriculteurice* (for *agricultrice*_fem_/*agriculteur*_masc_ ‘female/male farmer’), and *actifive* (for *active*_fem_/*actif*_masc_ ‘active’). The second case corresponds to word pairs ending in -*ine*_fem_/-*ain*_masc_ and -*ine*_fem_/-*in*_masc_ for which Borde proposes an epicene form ending in -*aine*: hence *copaine* and *cousaine* for the word pairs *copine*_fem_/*copain*_masc_ ‘female/male pal’ and *cousine*_fem_/*cousin*_masc_ ‘female/male cousin’, respectively. The third case concerns word pairs whose masculine singular written form ends with an unpronounced consonant that, by contrast, is pronounced in the feminine form: for example *candidat*_masc_ ‘male candidate’ (pronounced /kãdida/) vs. *candidate*_fem_ ‘female candidate’ (pronounced/kãdidat/). In this case Borde proposes that the final consonant be replaced in epicene forms by a close consonant: thus /t/ is replaced by /d/ in *patiende*, the epicene form corresponding to *patiente*_fem_/*patient*_masc_ ‘female/male patient’, /n/ is replaced by /m/ in *humaime*, the epicene form corresponding to *humaine*_fem_/*humain*_masc_ ‘female/male human’, and so on.

Similarly, Alpheratz (2018) proposes different morphemes for the production of new epicene nouns or inflected forms. The character *æ* (*a ligaturé*), unused in standard French, is suggested to replace the letter *e* to produce epicene forms for word pairs ending in -*ée*_fem_/-*é*_masc_, -*elle*_fem_/-*el*_masc_, -*ère*_fem_/-*er*_masc_, and -*erte*_fem_/-*ert*_masc_. The epicene forms corresponding to *aimée*_fem_/*aimé*_masc_ ‘loved’, *conseillère*_fem_/*conseiller*_masc_ ‘female/male counselor’, or *experte*_fem_/*expert*_masc_ ‘female/male expert’ are thus *aimæ*, *conseillær*, and *expært*, respectively. Alpheratz proposes to use the ending -*an* to produce epicene forms for word pairs ending in -*aine*_fem_/-*ain*_masc_ and -*ienne*_fem_/-*ien*_masc_, and, conversely, to use the ending *-aine* as an epicene marker for word pairs ending in -*ine*_fem_/-*in*_masc_, which gives the epicene forms *human*, *musician*, and *cousaine* for the word pairs *humaine*_fem_/*humain*_masc_ ‘female/male human’, *musicienne*_fem_/*musicien*_masc_ ‘female/male musician’, and *cousine*_fem_/*cousin*_masc_ ‘female/male cousin’. Alpheratz also suggests to extend the use of the epicene suffixes *-aire* and *-taire*, used in standard French, to produce epicene forms for word pairs ending in -*euse*_fem_/-*eur*_masc_, and *-trice*_fem_/-*teur*_masc_, respectively. The epicene forms *jouaire* and *autaire* thus complement the pairs *joueuse*_fem_/*joueur*_masc_ ‘female/male player’ and *autrice*_fem_/*auteur*_masc_ ‘female/male author’. Finally, the epicene forms of almost all other types of nouns and adjectives are produced by using the morpheme *x* at the end of the words (*x* for singular and *z* for plural). Hence for example the epicene forms *amix*, *venux*, *actix*, and *candidax* correspond to the word pairs *amie*_fem_/*ami*_masc_ ‘female/male friend’, *venue*_fem_/*venu*_masc_ ‘arrived’, *active*_fem_/*actif*_masc_ ‘active’, and *candidate*_fem_/*candidat*_masc_ ‘female/male candidate’, respectively.

A third party recently advocating the epistemological need for a systematic epicene language (Touraille and Allassonnière-Tang, 2023) proposes another epicene grammatical solution named “sex-blind French.” This solution is described in detail in Marsolier et al. (2023) and in Marsolier (2023) for its presentation as an additional grammatical gender. It is based on the observation that, in Spanish and in Italian, Romance languages that also possess feminine and masculine grammatical genders, feminine and masculine forms are often contrasted by the final vowels *a* and *o*, for example in the word pairs Spanish *buena*_fem_/*bueno*_masc_ ‘good’ or Italian *bambina*_fem_/*bambino*_masc_ ‘female/male child’. In written French, feminine and masculine forms are usually differentiated by an alternation between the vowel *e* and the zero morpheme (ø). Thus, *candidate*_fem_ ‘female candidate’ and *candidat*_masc_ ‘male candidate’ can be schematized as “lexical base + *e*” and “lexical base + ø,” respectively. In this solution, the epicene forms are marked by a vocalic alternation with the vowel *i* and are schematized as “lexical base + *i*” (hence the form *candidati* for the previous example). For the cases where the lexical bases differ in the feminine and masculine forms, for example in the word pairs *belle*_fem_/*beau*_masc_ ‘beautiful’ or *bonne*_fem_/*bon*_masc_ ‘good’, the lexical base of the feminine form is taken systematically to build the epicene form, thus giving the epicenes *belli* and *bonni*, rather than *beaui* and *boni*. The main exceptions to this general principle are the word pairs ending in *-trice*_fem_/-*teur*_masc_, for which the epicene ending *-teuri* is preferred. Of note, this simple solution with vowel *i* has been initially proposed in a short article published online by a student collective media of Québec (Martin, 2016). This proposal mentioned the creation in French of a “third, universal grammatical gender that could designate any person,” and suggested to use the vowel *i* as a marker for this new gender, called *épicène* ‘epicene’. In this proposition, feminine forms were also considered to be more suitable as a basis for the production of epicenes. However, to the best of our knowledge, Martin has not developed this idea with other works and his proposal has not been taken up by other publications, including those that provide an extensive list of epicene writing techniques (Alpheratz, 2018; La vie en queer, 2018; Lessard and Zaccour, 2018). The solution described above was therefore developed independently long before the authors became aware of Martin’s article (Touraille and Allassonnière-Tang, 2023).

### 1.3 Overview of the present study

As far as we know, the three proposals above are the only ones published, that each provide a complete and consistent scheme for a new, epicene, grammatical gender in French. These proposals involve very different processes, including consonantal or vocalic alternations, and some of them include the use of characters not found or rarely found in standard French, like *æ* and *ë*. Although these solutions are used in some communities and even in a few articles published in academic journals (e.g., (Ashley, 2019; Moron-Puech et al., 2020) for the solution described by Alpheratz), their learnability has not been addressed so far, even though it is crucial for assessing the probability that a new epicene grammatical gender is used and eventually integrated into standard French (Ashley, 2019). In the present study, we provide a first assessment of this aspect by means of an online translation test. For each of the three solutions, hereafter denoted A (for Alpheratz), B (for Borde), and I (according to its epicene gender marker), French-speaking participants were instructed that they would be trained to learn an “alien” language that does not mark sex/gender categories (these alien languages correspond to standard French where only gendered words referring to people are replaced by non-standard epicene forms). After a short learning-by-example phase, participants were required to translate into the alien language a set of 16 standard French sentences. The translations were analyzed as a function of several variables including the participants’ self-reported age and sex, the word categories and the solutions themselves. While all three solutions proved quickly learnable, participants’ responses with solution I achieved the highest accuracy score, in particular with regard to the production of non-standard epicene forms.

## 2 Materials and methods

### 2.1 Participants

For each solution (A, B, or I), we targeted a sample size of 40 online participants (50% self-identified females and 50% self-identified males) who were recruited through the platform Prolific.^1^ We restricted the participants to people whose first language was French and we excluded participants who had not correctly completed the learning phase or who had translated fewer than 14 sentences out of 16 in the test phase. A given experiment took on average 20 min to complete, and participants were rewarded with 4.5 £. Each participant only tested one of the three solutions. Participants’ self-reported age and sex were provided by Prolific as general information systematically requested from registered participants (the characteristics of the participants are presented in Supplementary Table S2). The median age of the groups was not significatively different (Wilcoxon’s rank test, *p* value = 0.62, 0.66, and 0.45 for comparison between groups for solutions A and B, A and I, and B and I, respectively).

### 2.2 Design

The experiment was designed using the PsychoPy software package^2^ that allows to run studies online with the repository and launch platform Pavlovia.^3^ Participants were first exposed to three introductory slides with the following messages (the original French text is available in Supplementary Text S1):

“Aliens speak a language that never indicates sex/gender. For words that have different masculine and feminine forms, for example: *il*/*elle*, *un*/*une*, *beau*/*belle*, *musicien*/*musicienne*, they have invented a third form that applies to all people, regardless of sex/gender.

On the other hand, these aliens use the standard forms of words when these do not indicate sex/gender. For example: *scénariste*, *libre*, *cosmonaute*, *les*, *votre*… And aliens always use the standard forms of words when these words refer to things (which have no sex/gender…). For example: *une belle bicyclette*.

The aliens therefore modify as few words as possible: they only change the words that mark sex/gender. As these aliens are altruistic, you try to master their language as best you can to foster an alliance. To help you practice, 16 sentences are first shown with translation, then a second series of 16 without translation.”

Following this introduction, which gives important cues regarding the parcimony of the solutions, participants were trained on 16 examples of translated sentences. For each example, the standard French sentence was presented on a first slide with its translation into the alien epicene language. The participant was again shown the standard French sentence on a second slide (without its translation), and was requested to write its translation. A third and final slide displayed the standard French sentence, its translation by the participant, and the correct translation. The training phase was immediately followed by the test phase which consisted in translating 16 new standard French sentences (one sentence per slide). No correction was provided in this part. To simplify the writing of answers for solution A, the character *æ* was systematically replaced by the couple of letters *ae*.

The sentences to be translated are listed in Supplementary Text S1. Each sentence from the test phase is associated with a sentence from the learning phase in that both sentences contain words whose translation into the alien epicene language requires the same changes. For example, the test sentence *une représentante suisse et le mécanicien français* ‘a Swiss representative and the French mechanic’ matches the learning sentence *la correspondante anglaise et un musicien belge* ‘the English correspondent and a Belgian musician’ as, in all solutions, the epicene forms of the words *représentante* and *correspondante* are produced by the same procedure (which gives *représentanx* and *correspondanx* for example in solution A); similarly for the words *mécanicien* and *musicien*, and so on. This design was intended to help participants: all types of epicene forms whose production was requested in the test phase were shown in a comparable context during the learning phase (see Supplementary Text S1 and Supplementary Table S3). We included in the test a large range of word types, so as to cover extensively the different procedures involved in producing epicene forms (Supplementary Table S3). Learning sentences were presented in the fixed order indicated in Supplementary Text S1, starting with simpler noun phrases and progressing to more complex ones. Test sentences were presented in random order.

### 2.3 Data preparation

The raw data corresponding to the participant response files provided by the Prolific platform are available on the Open Science Framework site.^4^ The participants translations of the 16 test sentences were processed as follows: punctuation signs, apostrophes, and extra spaces were removed, capital letters were replaced by lower-case letters, and the often misspelled *ç* character was replaced by a *c*. The 16 test sentences numbered a total of 120 words, hence an expected number of 4,800 words (120 times 40 participants) to analyze for each solution. Some participants forgot a few words or one sentence, so that the final number of words analyzed was 4,774, 4,780, and 4,766, for solutions A, B and I, respectively. Participants’ answers were manually reviewed and corrected for a few typos (mainly accents) that did not affect word endings (for example, *mecanician* instead of *mécanician*). This correction procedure modified 29, 53, and 35 words for solutions A, B and I, respectively, and eliminated errors that were not relevant to our analyses. Words were classified using the part-of-speech categories recommended by Straka and Straková (2017) for the Universal Dependencies framework (for details see^5^). The processed data, corresponding to 14,320 words, are shown in Supplementary Table S4.

### 2.4 Statistical analysis

All statistical analyses were performed with the R environment (R Core Team, 2017), using the packages *tidyverse* (Wickham et al., 2019) and *lme4* (Bates et al., 2015). The analyses scripts are available in Supplementary Text S1.

## 3 Results

### 3.1 Global analysis of accuracy

A correct translation of the test sentences required (i) to convert the nouns denoting persons and their satellite elements, when their forms are not epicene in standard French, into the non-standard, epicene form prescribed by the solution under test, and (ii) to leave all other words unchanged, be they words unaffected by gender agreement (verbs, conjunctions, etc.), or nouns and associated elements that do not refer to persons, or whose forms are epicene in standard French (e.g., *suisse* ‘Swiss’ or *disponible* ‘available’). Two types of “errors” (i.e., discrepancies between the observed and the expected translated forms) relevant to our analysis could be expected and were observed: (i) a specific, non-standard, epicene form was required but the participant produced either the standard, gendered form or an incorrect form; (ii) a word that should not be changed was altered into an incorrect form resembling the non-standard epicene forms specified by the solution.

We used generalized linear mixed models (Bates et al., 2015) to evaluate the effects of several variables on word translation accuracy. The response variable was a binary number indicating whether a given word had been correctly translated. Participants’ identifiers and words’ identifiers (the 16 test sentences contained a total of 120 words) were used as random effects. The final model included three fixed-effects variables: the participants self-identified sex/gender, the solution (A, B, or I), and the variable *change* classifying words into five levels: gendered words that refer to people corresponded to the level “person_gendered” and should be replaced by non-standard epicene forms, whereas words corresponding to the other four levels (“no_agreement,” for words like conjunctions or adverbs belonging to a part-of-speech (POS) category without gender agreement; “person_epicene,” for epicene words referring to people; “thing_epicene,” for epicene words referring to objects; “thing_gendered” for gendered words referring to objects) should not be changed. We did not consider both *POS* and *change* in the same model since the two variables are highly correlated. The age of the participants was included in the initial model but was removed as it did not show a significant effect (see details in Supplementary Text S1).

As shown in Table 1, the effects exhibited by the variable *change* were consistent with the definition of its levels. The level “thing_epicene” had no significant effect compared to the reference level “no_agreement,” which can be explained by the facts that in both cases the word should not be modified by the translation and this correct answer is easy to select: “no_agreement” words have no gender marker, and “thing_epicene” words should not be changed into a non-standard epicene form for two reasons, because they do not refer to people and because their form is epicene in standard French. The “thing_gendered” and “person_epicene” levels had strong negative effects on translation accuracy, which can be accounted for by the fact that in these cases only one criterion precludes a modification of the word, either the fact that the form is already epicene or the fact that it does not refer to people. Finally, the “person_gendered” level had the strongest negative effect, as expected since, in this case, not only the word had to be identified as requiring a change, but the correct change had to be implemented.

**Table 1:** Parameters of the fixed-effects variables of the generalized linear mixed model for all words.

	Estimate	Std. Error	*z* value	Pr(> |z|)	Significativity
(Intercept)	6.1833	0.3105	19.911	< 2e-16	***
sex [Male]	−0.7010	0.2004	−3.497	0.00047	***
solution [A]	−1.6669	0.2475	−6.735	1.64e-11	***
solution [B]	−1.3212	0.2476	−5.336	9.49e-08	***
change [person_epicene]	−2.2810	0.3727	−6.120	9.37e-10	***
change [person_gendered]	−4.4268	0.2776	−15.949	< 2e-16	***
change [thing_epicene]	−0.6176	0.8886	−0.695	0.48702	
change [thing_gendered]	−1.7062	0.3614	−4.721	2.35e-06	***

The results also show that self-identified male participants performed significantly worse than self-identified female participants and that translation accuracy was highly solution-dependent, with solutions A and B having a strong negative effect compared to solution I.

We examined whether the short learning-by-example phase had a significant effect on participants’ ability to translate test sentences for all solutions. Overall participants’ performance was measured as the proportion of correctly translated words. If we consider that in the absence of a learning phase, participants would simply translate the test sentences by copying them exactly, we get a baseline accuracy score equal to the proportion of words in the test sentences that must remain unchanged under translation. This baseline score is about 0.57 and fluctuates slightly according to the solutions specifications and the final number of words analyzed. We found that the participants’ performance was significantly higher than the baseline score for all three solutions (Supplementary Figure S1, Wilcoxon’s rank test, *p* value = 3 × 10^−8^, 3 × 10^−8^, and 2 × 10^−8^, for solutions A, B, and I, respectively).

### 3.2 Accuracy as a function of part-of-speech categories

**Figure 1:**
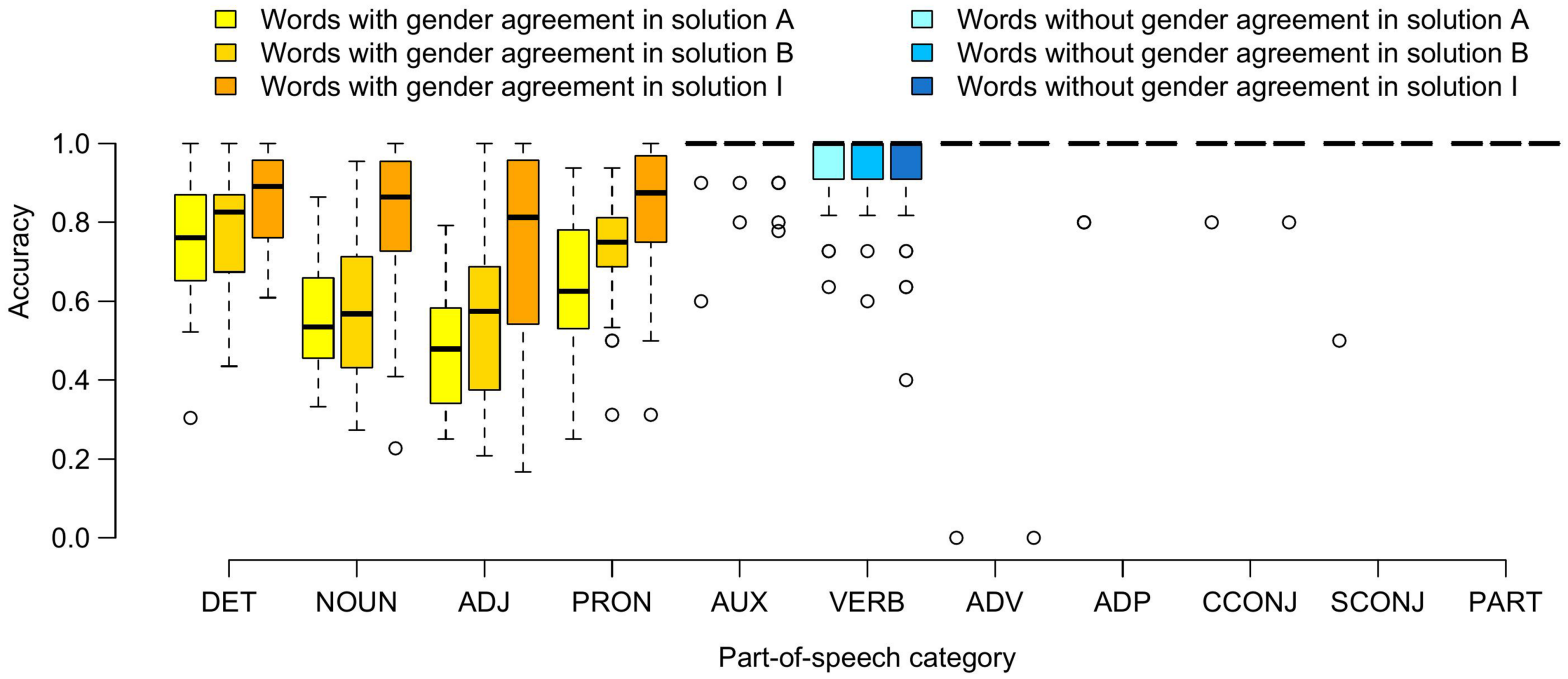
Participants’ accuracy as a function of part-of-speech categories and solutions. Each box plot represents the distribution, for a given part of speech, of the accuracy scores of the 40 participants who tested the indicated solution. DET, determiner; ADJ, adjective; PRON, pronoun; AUX, auxiliary; ADV, adverb; ADP, adposition; CCONJ, coordinating conjunction; SCONJ, subordinating conjunction; PART, particle.

Figure 1 shows participants’ performance by POS categories. Consistent with the previous analysis, the accuracy score for auxiliaries, adverbs, adpositions, conjunctions and particles (POS categories corresponding to the “no_agreement” level) was equal to 1 for the vast majority of participants and for all solutions. We found a higher number of incorrect forms for verbs and we noted that most of these forms included morphemes characteristic of the non-standard epicene words recommended by the solution studied: for example, *serrae* and *parlx* instead of *serra* ‘squeezed’ and *parla* ‘talked’ for solution A, *écoutë* and *vouluët* instead of *écouter* ‘listen’ and *voulut* ‘wanted’ for solution B, *serri* and *parli* instead of *serra* ‘squeezed’ and *parla* ‘talked’ for solution I. The proportions of these epicene-like forms over the total number of incorrect verb forms were equal to 17/26, 7/22, and 27/34 for solutions A, B, and I, respectively. We thus observed a limited “spillover” of non-standard epicene markers onto words devoid of gender agreement.

Accuracy scores for determiners, nouns, adjectives, and pronouns (POS categories with gender agreement) were lower and strongly dependent on the solution studied. In all cases, the participants’ scores obtained with solution I were significantly higher than those obtained with solutions A and B (Wilcoxon’s rank test for comparison between solution I and solutions A and B, respectively, *p* value = 0.003 and 0.003 for determiners, 2 × 10^−8^ and 3 × 10^−7^ for nouns, 4 × 10^−7^ and 5 × 10^−5^ for adjectives, 8 × 10^−7^ and 0.001 for pronouns). The scores for solutions A and B were not significantly different, except for pronouns (Wilcoxon’s rank test, *p* value = 0.9, 0.3, 0.2, and 0.004 for determiners, nouns, adjectives and pronouns, respectively). Results from generalized linear mixed models specifically built for each POS category also showed that translation accuracy with solution I was significantly higher than with solutions A and B in all cases (see details in Supplementary Text S1).

### 3.3 Analysis of words with gender agreement

We used another generalized linear mixed model to evaluate specifically the effects of variables on translation accuracy for words with gender agreement. The final model included four fixed-effects variables: the participants self-identified sex/gender, the solution type and the variables *change* and *POS* (see details in Supplementary Text S1).

In agreement with the previous analysis, we found that self-identified female participants performed significantly better than self-identified male participants (*p* value = 0.0006) and that the “person_gendered” category of the variable *change* had a strong negative effect (*p* value = 8 × 10^−6^) on translation accuracy. Pronouns were translated with a significantly higher accuracy than nouns (the reference category for the *POS* variable, *p* value = 0.009), whereas no significant difference in translation accuracy was observed between nouns and adjectives or between nouns and determiners (Supplementary Table S5).

**Figure 2:**
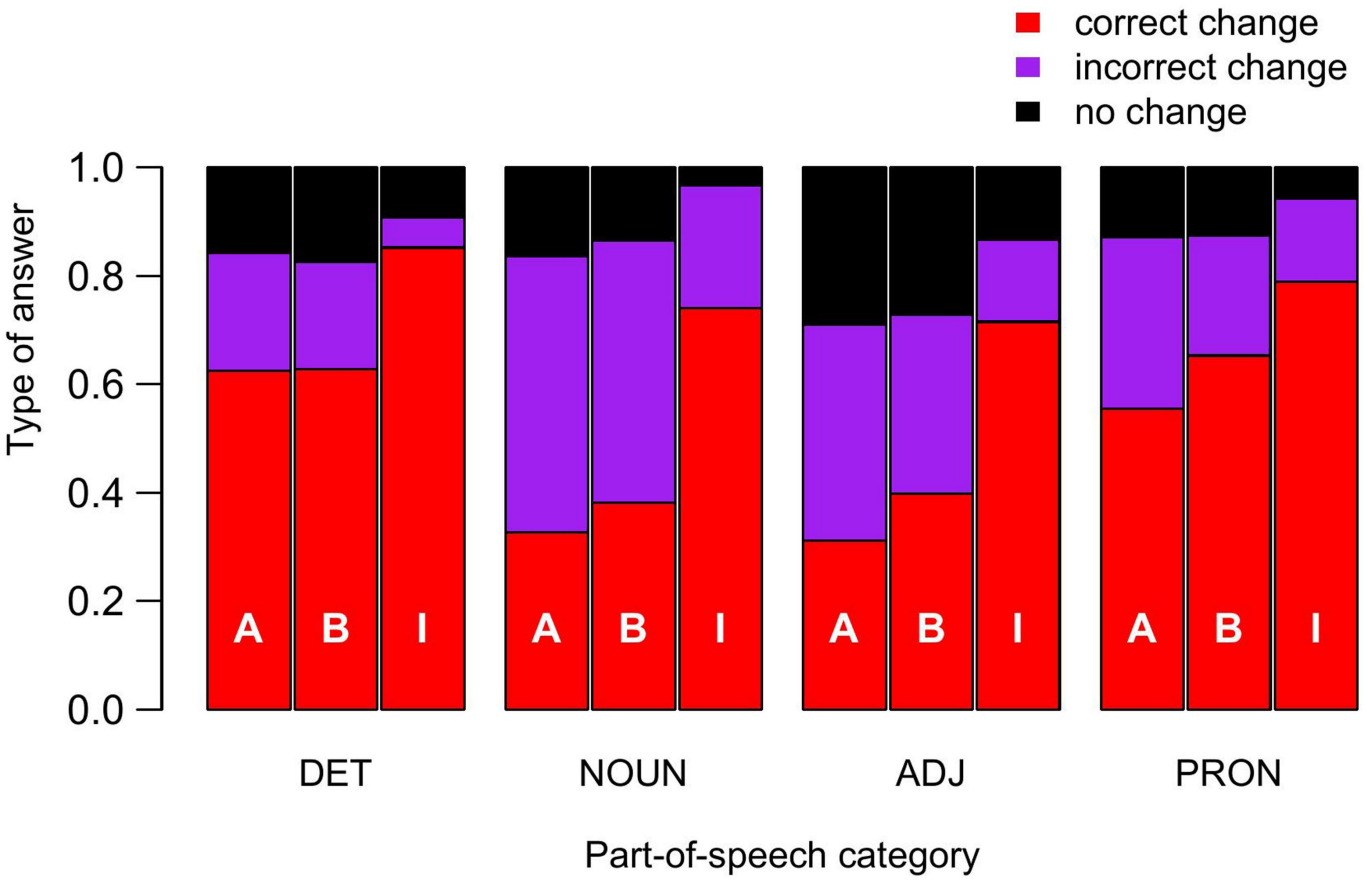
Type of responses as a function of POS categories and solutions. DET, ADJ and PRON are as in Figure 1. The solutions are indicated by the white letters A, B and I on the bars.

The translations of gendered words referring to people can be classified into three types of response: (i) no change, when the gendered form of the test sentence has simply been copied, (ii) a change to the non-standard epicene form specified by the solution (correct change), or (iii) a change to an incorrect form. Figure 2 shows the proportions of responses corresponding to these options, by POS category and solution. Retaining a gendered word referring to people in the translated sentences can be explained either by the fact that the participants do not realize that the word needs to be changed, or by the fact that they do not know which form to replace it with. A comparison of the results for the three solutions suggests that this last possibility is probably the most frequent for solutions A and B. Indeed, since the same test sentences are used to analyze all three solutions, the probability of not identifying a word as a form to be changed is *a priori* the same for all three groups of participants. This probability therefore admits as an upper bound the minimum value of the proportions of unchanged words across the three solutions for a given POS category. In all cases, this minimum value corresponds to the proportion of unchanged words for solution I, that is 0.092, 0.032, 0.13, and 0.057 for determiners, nouns, adjectives and pronouns, respectively. By subtracting these minimum values from the proportions of words that were not changed for solutions A and B, we should logically obtain the proportions of words that were not changed due to ignorance of the form to be used, and these cases represent the majority of gendered words referring to people that remained unchanged in the translations with solutions A and B.

**Figure 3:**
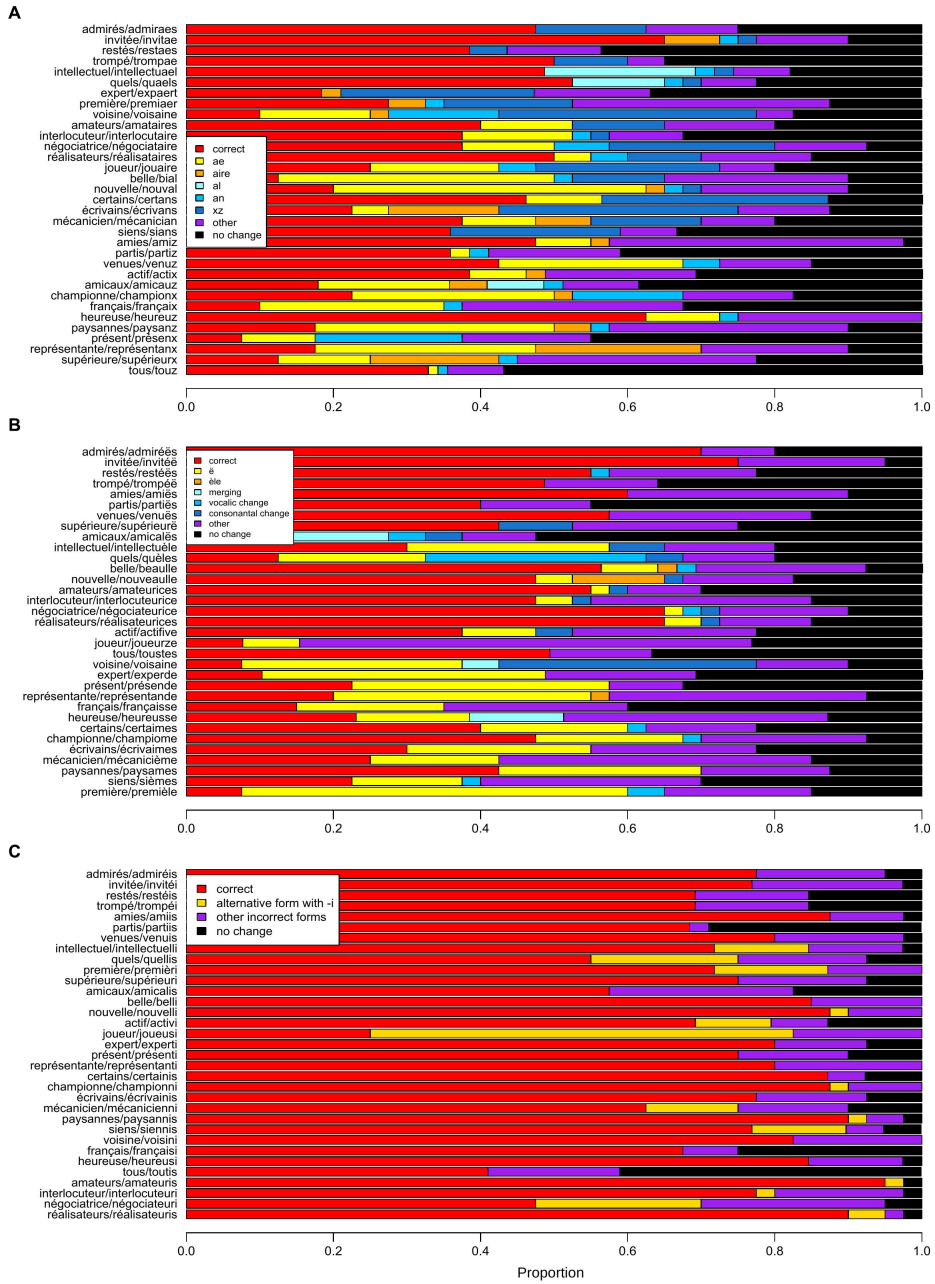
Proportions of unchanged, correctly and incorrectly modified forms produced by the participants for the gendered nouns and adjectives that were to be replaced by a non-standard epicene form according to solutions A **(A)**, B **(B)** and I **(C)**. The gendered words and the corresponding epicene forms are indicated on the left of the graphs. Words are sorted according to their markers for the epicene gender in **(A)** and **(B)**. In **(A)**, markers are arranged in this order: -*ae*, -*aine*, -*aire*, -*al*, -*an*, and -*x*/-*z*. In **(B)** the order is: epicene forms in -*ë*, in *-èle*, epicene forms built by merging, by vocalic change and by consonantal change. As -*i* is the only epicene marker of solution I, words were sorted in **(C)** according to the ending of their standard forms.

Finally, we examined the forms corresponding to incorrect changes of gendered nouns and adjectives that should have been changed into the non-standard epicene forms specified by each solution. Solutions A and B propose different morphemes or processes as markers of the epicene gender and an incorrect form often incorporate one of the epicene markers recommended by the solution but not the one that should be used in that case. For example, *voisine*_fem_ ‘female neighbor’ should be changed into the epicene *voisaine* according to solution A, but the participants have also produced the forms *voisinx*, *voisinz*, *voisinae*, *voisan* and *voisaire*, using the epicene markers *-x*, -*z*, -*ae*, -*an*, and -*aire* that are recommended by solution A for other words. These incorrect forms were manually categorized according to the inappropriate epicene marker they exhibited (when that was the case). The results of this classification are shown in Figure 3 (see also Supplementary Tables S6–S8 for the list of these forms). Concerning solution A, the two epicene markers that were the most frequently used in incorrect forms are the two simplest ones, -*ae* and -*x*/-*z* (157 and 121 words, respectively, compared to the third best, the marker -*an*, with 43 words). Similarly, the marker -*ë* was by far the most used in incorrect forms for solution B (177 words compared to the second best process, consonantic change, with 32 words). Solution I has only one epicene marker, the vowel *i*, but epicene forms can be derived either from feminine or masculine forms. As shown in Figure 3C, a substantial proportion of erroneous forms were due to an alternative derivation of the epicene form, from the masculine when the feminine was recommended, and *vice-versa*.

## 4 Discussion

The problem with the generic use of the masculine coupled with the challenge of sex categorization itself has led to proposals for new epicene grammatical genders in many languages around the world. French is no exception to this trend and we have identified three such proposals that differ in many ways: Alpheratz’s and Borde’s solutions use a variety of epicene markers while solution I only uses the vowel *i*; in contrast with solution I, Alpheratz’s and Borde’s solutions employ non-standard or rarely used characters (*æ* and *ë*, respectively); in speech, epicene forms in Borde’s solution and in solution I are systematically differentiated from the corresponding feminine and masculine when these two differ from one another, whereas many epicene forms in Alpheratz’s solution are pronounced like the masculine (e.g., almost all epicene words ending in -*x* and -*z*); finally, epicene forms in solution I always differ in speech from the feminine and the masculine, whereas epicene, feminine and masculine forms can be homophonous in Alpheratz’s and Borde’s solutions.

We provide here a first assessment of the short-term learnability of these proposals: after a ~ 12 min learning phase, participants were asked to translate 16 standard French sentences into one of the epicene languages defined by these solutions. The translation of a given word had to correspond exactly to the form specified by the solution to be considered correct: a non-standard form built with an epicene marker different from the one recommended by the solution for that case was considered incorrect. This choice, which simplified the estimate of accuracy scores, was motivated by the fact that, as discussed by Ashley (2019), a rule-based, systematic approach for a new epicene grammatical gender makes it more likely to be one day integrated into standard French. We thus decided to evaluate the solutions using this strict criterion.

We found that all three solutions are quickly learnable, with participants’ global accuracy scores significantly higher than the baseline. Participants’ responses with solution I achieved a significantly higher accuracy score than the responses with the solutions proposed by Alpheratz and Borde for all categories of words with gender agreement. As discussed above, the lower proportions of correct forms observed with Alpheratz’s and Borde’s solutions were due to both a higher number of non-standard incorrect forms and to fewer replacements of the gendered forms, probably caused by the ignorance of the form required by the solution rather than by the failure to identify gendered words referring to people as forms to be replaced.

We also noted that the performance of the self-identified female participants was significantly higher than that of the self-identified male participants. This discrepancy could be due either to the object of the test (women could be more interested in learning an epicene language) or to its procedures. Indeed, the test relies heavily on the ability to write precise word forms and in France there is a wide gap between girls and boys in spelling and foreign language skills throughout the school years (Chabanon and Steinmetz, 2018), which could persist afterwards and be reflected here. However, we cannot exclude that other variables like the affiliation with specific social groups could affect participants’ performance, so this result remains to be investigated further.

Obviously this study presents several limitations. First, it assesses only the learnability of the solutions as far as writing is concerned. Given that, as mentioned above, the three solutions have also very different oral characteristics, their speaking learnability could vary considerably from one another, and from their writing learnability. Second, the learning phase was short and based on examples, not rules. Third, the participants’ proficiency in the epicene solutions was tested immediately after the learning phase, and its medium- and long-term permanence was not assessed. Finally an important limitation of this study is the fact that the set of participants was not representative of the global French population since they were recruted through an online international platform, which restricted the sampling to people familiar with English and with computers. These features may have impacted our results in various ways. Thus better accuracy scores could probably be achieved with in-person training, a more extended learning phase or the statement of explicit rules. It is more difficult to assess how the use of an online platform might have influenced performance: the participants recruted in this way are obviously more at ease with computers and with English than the general French population, but whether that makes them more proficient at learning new morphological features is unclear. Two points can be made regarding the relevance of the study methodology. First, informal linguistic changes generally propagate through occasional examples of novelties read or heard, rather than through rules statement or lengthy explanations, hence the relevance of a short learning-by-example phase to test the solutions’ learnability under more realistic conditions. Second, even if participants recruted through online platforms were statistically better or worse at learning new words than the average French population, the main objective of the article was to compare the solutions’ learnability, and it seems plausible that their learnability ranking should be independent of the participants’ overall linguistic competence. Nevertheless the above-mentioned limitations remain and addressing them will be an objective for future studies. New directions of research could include assessing the solutions’ learnability in speech, evaluating the long-term persistence of skills acquired by participants, both in writing and in speech, and targeting a more diversified set of participants by varying recruitment procedures (advertisements in local newspapers or more specialized journals, presentations in schools or homes for the elderly, etc.). Finally, another aspect of gender inclusivity in French, not addressed in this study, concerns gendered noun pairs with distinct radicals (e. g. *sœur*/*frère* ‘sister’/‘brother’). The new epicene nouns that have been suggested to complement these pairs usually exhibit yet another radical (*adelphe* ‘sibling’ is one such proposal for *sœur*/*frère*). An analysis of the parameters that can influence the choice between various epicene correspondents for these pairs would be a valuable supplement to the present study.

With all the caveats previously mentioned in mind, the present study establishes the principle of vowel alternation with a final *i* as a way to create a new epicene grammatical gender in French that is easy to learn, at least in writing and on a short-term basis, and relative to Alpheratz’s and Borde’s proposals. The advantage of short morphemes as epicene markers over more complex processes (e.g., vocalic or consonantal changes) is also illustrated by the fact that erroneous non-standard forms produced by participants with Alpheratz’s and Borde’s solutions are predominantly derived using the simplest epicene markers of each solution, -*ae* and -*x*/-*z*, and -*ë*, respectively. Interestingly, the principle of final vowel alternation for building an epicene grammatical gender is already largely experimented among both Spanish- and Italian-speakers: in Spanish, final -*e* is one of the most popular “inclusive” gender markers (Papadopoulos, 2022) and in Italian the vowel ə (called *schwa*) has even been adopted by a publishing house (Sulis and Gheno, 2022). These observations could be partly accounted for by the fact that the alternation of final vowels is a feature of Romance languages that often differentiates feminines and masculines so that its extension to produce forms of a new grammatical gender seems fairly intuitive.

Regardless of which morphological solution would eventually be preferred, the availability of a new epicene grammatical gender represents a conceptual option distinct from the current “inclusive” option. Let us remember that the so-called inclusive solutions are the only ones that have been discussed in France in recent years, and the only linguistic changes that seem to have any chance of prevailing, at least in academic circles. As detailed in the introduction, the current “inclusive” option, which aims to move away from the hegemony of the masculine grammatical gender in the French language, also overtly tends to make sex categories appear somehow “necessary,” which is a serious epistemic question. Moreover, this option is often incoherent in practice, with scientific texts mixing feminizing strategies with epicene strategies such as the non-binary pronoun *iel* (equivalent to *they* or *ze* in English). A new epicene grammatical gender, which would provide a genuine and coherent epicene mode of expression, would allow to overcome not only the hegemony of the masculine grammatical gender, but also the (reputedly unescapable) sex/gender grammatical categorization. From a scientific point of view, as Touraille and Allassonnière-Tang (2023) have recently argued, an epicene grammatical gender represents an epistemological break with common sense, which is in fact much needed in sociological and gender studies research, and in science in general. From a societal point of view, the epicene option is particularly relevant for non-binary people, as it would allow anyone who so wishes to speak French without being systematically identified by “their” sex/gender, whether in everyday communication or in more official speeches and writings. Of note, this would notably improve translations both from languages without a sex-based grammatical gender and from texts that voluntarily avoid gender markers. The epicene option is also of interest for trans people, who suffer the most from linguistic misgendering in everyday life. Finally, and maybe most crucially, it would offer a kind of cultural detachment from sex categorization that would be particularly of help to intersex communities currently fighting for the legal right not to have their sex surgically “fixed” in infancy (Karkazis, 2008). Let us not forget that the traumatic surgical practices of “sex assignment” and eugenic policies on viable intersex fetuses depend largely on the female/male categories with which children are supposed to closely identify, and in which they are obligatorily raised and visually differentiated in Western societies. For everyone, systematic use of epicene forms would eliminate the linguistic distinction between a child’s parents or relatives (in a school, social or legal context), would promote non-discriminatory policies for recruitment, promotion, etc. in a professional environment, and would also contribute to lessen anti-LGBT+ hate speech, which is particularly acute in children’s cultures. It may help in a general way to promote a more egalitarian, less sexually-oriented perception of human beings.

In the face of the reactions that have been systematically opposed to the “inclusive” solutions in French (typically the problem of illegibility and redundancy in spoken language), a new epicene grammatical gender is a formally parsimonious option to fight the generic value of the masculine gender. Moreover, its impact on French practices and morphology is quite limited since new epicene forms appear only for nouns of persons and their satellite elements, and only when their forms are not epicene in standard French. The feminine and masculine forms remain unchanged for all nouns designating objects or concepts and their satellite elements, which correspond to the major part of speech or texts.

A plausible scenario for the diffusion of this new epicene grammatical gender can be sketched as follows. As a scientific conceptual tool, it would first be necessary to convince the scientific community (and specifically scholars in gender studies) of the importance of an epistemological break with ordinary sex categories. This could be achieved through discussion, but also through the example of using new grammatical forms in scientific publications (e. g. in [Touraille and Allassonnière-Tang, 2023]). Concerning social change, the first step could be the establishment of a consensus about preferred forms among its proponents (mostly non-binary communities nowadays). This is where our study seems particularly relevant as an example of comparing available solutions for desirable parameters (learnability in the present case), and trying to determine the best option. Once a consensus has been reached [as called by Ashley (2019)], these specific epicene forms could be proposed for use in legal and administrative texts, as French jurist scholars have already started to do (Moron-Puech et al., 2020). These new forms will eventually get noticed and trigger a reaction that could turn into a general debate among Francophones, offering some publicity and the opportunity to explain their *raison d’être* and construction rules. At that point, resistance will emerge with certainty, as is already the case today with so-called inclusive writing. Indeed, it can be noticed that proposals to introduce a new epicene grammatical gender are usually met with fierce resistance from political institutions around the world [e.g. (Sulis and Gheno, 2022)]. While it is beyond the scope of this article to present a thorough analysis of the causes of this resistance and strategies to overcome it, it is worth noting, as pointed out in the introduction, that French people and institutions are particularly conservative on this subject. The battle between supporters and opponents of the new grammatical gender could last some years or some decades [it took 35 years for the Académie française to stop opposing the feminine forms of profession names (Académie française, 2019)], and so the socio-political context will be decisive.

## Data availability statement

The datasets presented in this study can be found in online repositories. The names of the repository/repositories and accession number(s) can be found in the article/Supplementary material.

## Ethics statement

Ethical review and approval was not required for the study of human participants in accordance with the local legislation and institutional requirements. Written informed consent for participation was not required from the participants in accordance with the national legislation and institutional requirements.

## Author contributions

M-CM: Conceptualization, Data curation, Formal analysis, Investigation, Software, Writing – original draft, Writing – review & editing, Methodology. PT: Conceptualization, Investigation, Writing – review & editing. MA-T: Conceptualization, Methodology, Writing – original draft, Writing – review & editing.

## Footnotes

^1^https://www.prolific.co/

^2^https://www.psychopy.org/

^3^https://pavlovia.org/

^4^https://osf.io/8v4cy/

^5^https://universaldependencies.org/u/pos/index.html
