# Supplementary material for: Vowel alternation with final i offers an easy-to-learn morphological option for a sex-blind grammatical gender in French
Source: Front Psychol. 2024 Feb 22;15:1310475. doi: 10.3389/fpsyg.2024.1310475 (PMC10938390; doi:10.3389/fpsyg.2024.1310475)
Supplement: Supplementary file 1 [file Data_Sheet_1.ZIP › Data_Sheet/Data Sheet 1.docx]

**Supplementary Table S1.** Overview of the epicene forms proposed by solutions A, B, and I. “F/m” stands for “feminine/masculine”. Plural markers or forms are shown in brackets if different from the singular: *(s)* and *(x)* indicate a final *s* or *x*; plural forms that cannot be derived from singular forms by addition of a final *s* or *x* are shown in full. For solution A, the epicene forms including the *æ* character are highlighted in gold, the epicene forms produced with the *-aire(s)* and *-taire(s)* suffixes are highlighted in blue, and the epicene forms produced with the suffixes *-al(-auz)*, *-an(s)*, *-ian(s)*, and *-aine(s)* are highlighted in red. All other epicene forms (with the exception of some pronouns and determiners) are marked by a final *x* or *z*. For solution B, the epicene forms including the *ë* marker are highlighted in light gold, the epicene forms ending in *-èle(s)* and *-aine(s)* are highlighted in orange and red, respectively, and the epicene forms that are produced by merging the masculine and feminine endings (in that order) are highlighted in green. All other epicene forms (with the exception of some pronouns and determiners) are marked by a consonantic change compared to the feminine and masculine forms. For solution I, almost all epicene forms are produced by replacing the final *e* or *a* (in determiners like *la*_fem_ ‘the’ or *ma*_fem_ ‘my’) of feminine forms by an *i*. Exceptions are highlighted in gold. Although the joint form of the indirect object complement personal pronoun *lui(leur)* is identical for female and male referents and is used as an epicene form in solutions B and I, solution A proposes to use *lu(leur)* instead, which accounts for its presence in the table. For solutions A and B, a question mark indicates epicene forms that are expected given the proposed production rules, but that are not attested in the examples provided by (Alpheratz, 2018) or (Borde, 2016).

**A. Nouns and adjectives (including past participles)**

| Word type | F/m ending | Solution A (Alpheratz) | Solution B (Borde) | Solution I | Translation |
| --- | --- | --- | --- | --- | --- |
| Homophonous feminine and masculine forms | *-ée(s)*/*-é(s)* | *-æ(s)*: *adoræ(s)* | *-éë(s)*: *adoréë(s)* | *-éi(s)*: *adoréi(s)* | ‘adored’ |
|  | *-ie(s)*/*-i(s)* | *-ix(-iz)*: *amix(amiz)* | *-ië(s)*: *amië(s)* | *-ii(s)*: *amii(s)* | ‘friend’ |
|  | *-ue(s)*/*-u(s)* | *-ux(-uz)*: *venux(venuz)* | *-uë(s)*: *venuë(s)* | *-ui(s)*: *venui(s)* | ‘arrived’ |
|  | *-elle(s)*/*-el(s)* | *-æl(s)*: *officiæl(s)* | *-èle(s)*: *officièle(s)* | *-elli(s)*: *officielli(s)* | ‘official’ |
|  | *-ile(s)*/*-il(s)* | *-ilx(-ilz)*: *subtilx(subtilz)* | *-ilë(s)*: *subtilë(s)* | *-ili(s)*: *subtili(s)* | ‘subtil’ |
|  | *-ole(s)*/*-ol(s)* | *-olx(-olz)*: *Espagnolx(Espagnolz)* | *-olë(s)*: *Espagnolë(s)* ? | *-oli(s)*: *Espagnoli(s)* | ‘Spanish’ |
|  | *-ulle(s)*/*-ul(s)* | *-ulx(-ulz)*: *nulx(nulz) ?* | *-ulë(s)*: *nulë(s)*? | *-ulli(s)*: *nulli(s)* | ‘null’ |
|  | *-ère(s)*/*-er(s)* | *-ær(s)*: *chær(s)* | *-erë(s)*: *cherë(s)*? | *-èri(s)*: *chèri(s)* | ‘dear’ |
|  | *-eure(s)*/*-eur(s)* | *-eurx(-eurz)*: *docteurx(docteurz)* | *-eurë(s)*: *docteurë(s)* | *-euri(s)*: *docteuri(s)* | ‘doctor’ |
| Feminine ending in  *-le(s)* and masculine ending in *-al(aux)*,  *-eau(x)*, or *-ou(s)* | *-ale(s)*/*-al(aux)* | *-alx/-auz*: *amicalx(amicauz)* | *-alë(s)*: *amicalë(s)* | *-ali(s)*: *amicali(s)* | ‘friendly’ |
|  | *-elle(s)*/*-eau(x)* | *-al(-auz)*: *nouval(nouvauz)*; *bial(biauz)* | *-eaulle(s)*: *nouveaulle(s)*; *beaulle(s)* | *-elli(s)*: *nouvelli(s)*;  *belli(s)* | ‘new’ ; ‘beautiful’ |
|  | *-olle(s)*/*-ou(s)* |  | *-oulle(s)*: *foulle(s)* | *-olli(s)*: *folli(s)* | ‘mad’ |
| Different final consonants for f/m forms | *-ive(s)*/*-if(s)* | *-ix(-iz)*: *actix(actiz)* | *-ifive(s)*: *actifive(s)* | *-ivi(s)*: *activi(s)* | ‘active’ |
|  | *-euse(s)*/*-eur(s)* | *-aire(s)*: *jouaire(s)* | *-eurze(s)*: *joueurze(s)* | *-eusi(s)*: *joueusi(s)* | ‘player’ |
| Feminine ending in [d] | *-ande(s)*/*-and(s)* | *-anx(-anz)*: *frianx(frianz)* | *-ante(s)*: *friante(s)* | *-andi(s)*: *friandi(s)* | ‘fond’ |
|  | *-arde(s)*/*-ard(s)* | *-arx(-arz)*: *Lombarx(Lombarz)* | *-arte(s)*: *Lombarte(s)* | *-ardi(s)*: *Lombardi(s)* | ‘Lombard’ |
| Feminine ending in [t] | *-ate(s)*/*-at(s)* | *-ax(-az)*: *avocax(avocaz)* | *-ade(s)*: *avocade(s)* | *-ati(s)*: *avocati(s)* | ‘lawyer’ |
|  | *-ête(s)*/*-êt(s)* | *-êx(-êz)*: *prêx(prêz)*? | *-êde(s)*: *prêde(s)* | *-êti(s)*: *prêti(s)* | ‘ready’ |
|  | *-ette(s)*/*-et(s)* | *-ex(-ez)*: *muex(muez)* ? | *-ède(s)*: *muède(s)* | *-etti(s)*: *muetti(s)* | ‘mute’ |
|  | *-ite(s)*/-*it(s)* | *-ix(-iz)*: *contrix(contriz)* | *-ide(s)*: *contride(s)*? | *-iti(s)*: *contriti(s)* | ‘contrite’ |
|  | *-ante(s)*/*-ant(s)* | *-anx(-anz)*: *confianx(confianz)* | *-ande(s)*: *confiande(s)* | *-anti(s)*: *confianti(s)* | ‘confident’ |
|  | *-ente(s)*/*-ent(s)* | *-enx(-enz)*: *insolenx(insolenz)* | *-ende(s)*: *insolende(s)* | *-enti(s)*: *insolenti(s)* | ‘insolent’ |
|  | *-erte(s)*/*-ert(s)* | *-ært(s)*: *expært(s)* | *-erde(s)*: *experde(s)*? | *-erti(s)*: *experti(s)* | ‘expert’ |
| Feminine ending in [n] | *-ane(s)*/*-an(s)* | *-anx(-anz)*: *partisanx(partisanz)* | *-ame(s)*: *partisame(s)* | *-ani(s)*: *partisani(s)* | ‘partisan’ |
|  | *-anne(s)*/*-an(s)* | *-anx(-anz)*: *paysanx(paysanz)* | *-ame(s)*: *paysame(s)* | *-anni(s)*: *paysanni(s)* | ‘farmer’ |
|  | *-aine(s)*/*-ain(s)* | *-an(s)*: *human(s)* | *-aime(s)*: *humaime(s)* | *-aini(s)*: *humaini(s)* | ‘human’ |
|  | *-ienne(s)*/*-ien(s)* | *-ian(s)*: *musician(s)* | *-ième(s)*: *musicième(s)* | *-ienni(s)*: *musicienni(s)* | ‘musician’ |
|  | *-ine(s)*/*-in(s)* | *-aine(s)*: *cousaine(s)* | *-aine(s)*: *cousaine(s)* | *-ini(s)*: *cousini(s)* | ‘cousin’ |
|  | *-onne(s)*/*-on(s)* | *-onx(onz)*: *mignonx(mignonz)* | *-ome(s)*: *mignome(s)* | *-onni(s)*: *mignonni(s)* | ‘cute’ |
| Feminine ending in [ʁ] and masculine ending in [e] | *-ère(s)*/*-er(s)* | *-ær(s)*: *conseillær(s)* | *-èle(s)*: *conseillèle(s)* | *-èri(s)*: *conseillèri(s)* | ‘advisor’ |
| Feminine ending in [z] | *-aise(s)*/*-ais* | *-aix(aiz)*: *Françaix(Françaiz)* | *-aisse(s)*: *Françaisse(s)* | *-aisi(s)*: *Françaisi(s)* | ‘French’ |
|  | *-oise(s)*/*-ois* | *-oix(-oiz)*: *Danoix(Danoiz)* | *-oisse(s)*: *Danoisse(s)* | *-oisi(s)*: *Danoisi(s)* | ‘Danish’ |
|  | *-euse(s)*/*-eux* | *-euz*: *heureuz* | *-eusse(s)*: *heureusse(s)* | *-eusi(s)*: *heureusi(s)* | ‘happy’ |
| Special cases | *-esse(s)*/*-e(s)* |  | *-èse(s)*: *hôtèse(s)* | *-e(s)*: *hôte(s)* | ‘guest’ |
|  | *-trice(s)*/*-teur(s)* | *-taire(s)*: *autaire(s)* | *-teurice(s)*: *auteurice(s)* | *-teuri(s)*: *auteuri(s)* | ‘author’ |

**B. Pronouns and determiners**

| Word type | F/m forms | Solution A (Alpheratz) | Solution B (Borde) | Solution I | Translation |
| --- | --- | --- | --- | --- | --- |
| Personal pronoun | *elle(s)*/*il(s)* (subject) | *al(s)* | *iel(s)* | *iel(s)* | ‘she/he(they)’ |
|  | *la(les)*/*le(s)* (direct object complement) | *lu(les)* | *lea(les)* | *li(les)* | ‘her/him(them)’ |
|  | *lui(leur)* (indirect object complement, joint form) | *lu(leur)* | *lui(leur)* | *lui(leur)* | ‘her/him(them)’ |
|  | *elle(s)*/*lui(eux)* (indirect object complement, disjunctive form) | *al(auz)* | *ellui(eulles)* | *iel(s)* | ‘her/him(them)’ |
| Demonstrative pronoun | *celle(s)*/*celui(ceux)* | *céal(çauz)* | *cellui(ceulles)* | *celli(s)* | ‘the one(s)’ |
| Possessive pronoun | *la(les) mienne(s)*/*le(s) mien(s)* | *lu(les) mian(s)* | *lea(les) mième(s)* | *li(les) mienni(s)* | ‘mine’ |
| Relative and interrogative pronouns | *laquelle(lesquelles)*/*lequel(lesquels)* | *luquæl(lesquæls)* | *leaquèle(lesquèles)* | *liquelli(lesquellis)* | ‘which’ or ‘which  one(s)’ |
| Definite article | *la(les)*/*le(s)* | *lu(les)* | *lea(les)* | *li(les)* | ‘the’ |
|  | *à la(aux)*/  *au(x)* | *à lu(aux)* | *aua(aux)* | *à li(aux)* | ‘to the’ |
|  | *de la(des)*/  *du(des)* | *de lu(des)* | *dua(des)* | *de li(des)* | ‘of the’ |
| Indefinite article | *une(des)*/*un(des)* | *an(des)* | *eune(des)* | *uni(des)* | ‘a’ or ‘an’ |
| Demonstrative determiner | *cette(s)*/*ce(s)* | *cex(cez)* ou *cæ(s)* ? | *cède(s)* | *cetti(s)* | ‘this’ or ‘that’ |
| Possessive determiner | *ma(mes)*/*mon(mes)* | *mu(mes)* | *man(mes)* | *mi(mes)* | ‘my’ |
|  | *ta(tes)*/*ton(tes)* | *tu(tes)* | *tan(tes)* | *ti(tes)* | ‘your’ |
|  | *sa(ses)*/*son(ses)* | *su(ses)* | *san(ses)* | *si(ses)* | ‘her’ or ‘his’ |
| Interrogative and exclamative determiner | *quelle(s)*/*quel(s)* | *quæl(s)* | *quèle(s)* | *quelli(s)* | ‘what’ or ‘which’ |
| Indefinite determiner or pronoun | *aucune*/*aucun* | *aucan* | *auqueune* | *aucuni* | ‘none’ |
|  | *chacune*/*chacun* | *chacan* | *chaqueune* | *chacuni* | ‘each’ |
|  | *quelqu’une(quelques unes)*/  *quelqu’un(quelques uns)* | *quelqu’an(quelques ans)* | *quelqu’eune*  *(quelques eunes?)* | *quelqu’uni(quelques unis)* | ‘someone’ |
|  | *toute(s)*/*tout(tous)* | *toutx(touz)* | *toude(toustes)* | *touti(s)* | ‘all’ |
|  | *certaine(s)*/*certain(s)* | *certan(s)* | *certaime(s)* | *certaini(s)* | ‘some’ or ‘certain’ |

**Supplementary Table S3.** List of the nouns denoting persons and their satellite elements that are included in the learning and test sentences. All these forms should be converted into non-standard epicene forms by the participants, except those indicated as « epicene » (bottom line of each table). “F/m” stands for “feminine/masculine”. The structure of Tables S1 and S3 is identical, allowing to identify the types of words whose conversion to non-standard epicene forms has been tested.

**A. Nouns and adjectives (including past participles)**

| Word type | F/m endings | Learning sentences | Test sentences |
| --- | --- | --- | --- |
| Homophonous feminine and masculine forms | *-ée(s)*/*-é(s)* | *comblée*, *rassurés*, *guidés* | *invitée*, *admirés*, *trompé*, *restés* |
|  | *-ie(s)*/*-i(s)* | *suivies*, *amis* | *amies*, *partis* |
|  | *-ue(s)*/*-u(s)* | *inconnues* | *venues* |
|  | *-elle(s)*/*-el(s)* | *ponctuel*, *sensationnels* | *intellectuel* |
|  | *-ile(s)*/*-il(s)* |  |  |
|  | *-ole(s)*/*-ol(s)* |  |  |
|  | *-ulle(s)*/*-ul(s)* |  |  |
|  | *-ère(s)*/*-er(s)* |  |  |
|  | *-eure(s)*/*-eur(s)* | *meilleure* | *supérieure* |
| Feminine ending in *-le(s)* and masculine ending in *-al(aux)*,  *-eau(x)*, or *-ou(s)* | *-ale(s)*/*-al(aux)* | *égaux* | *amicaux* |
|  | *-elle(s)*/*-eau(x)* | *nouvelle*, *belle* | *nouvelle*, *belle* |
|  | *-olle(s)*/*-ou(s)* |  |  |
| Different final consonants for f/m forms | *-ive(s)*/*-if(s)* | *sportif* | *actif* |
|  | *-euse(s*)/*-eur(s)* | *voleur* | *joueur* |
| Feminine ending in [d] | *-ande(s)*/*-and(s)* |  |  |
|  | *-arde(s)*/*-ard(s)* |  |  |
| Feminine ending in [t] | *-ate(s)*/*-at(s)* |  |  |
|  | *-ête(s)*/*-êt(s)* |  |  |
|  | *-ette(s)*/*-et(s)* |  |  |
|  | *-ite(s)*/*-it(s)* |  |  |
|  | *-ante(s)*/*-ant(s)* | *correspondante* | *représentante* |
|  | *-ente(s)*/*-ent(s)* |  | *présent* |
|  | *-erte(s)*/*-ert(s)* | *découvert* | *expert* |
| Feminine ending in [n] | *-ane(s)*/*-an(s)* | *partisanes* |  |
|  | *-anne(s)*/*-an(s)* |  | *paysannes* |
|  | *-aine(s)*/*-ain(s)* | *humains* | *écrivains* |
|  | *-ienne(s)*/*-ien(s)* | *musicien* | *mécanicien* |
|  | *-ine(s)*/*-in(s)* | *cousine* | *voisine* |
|  | *-onne(s)*/*-on(s)* | *mignonne* | *championne* |
| Feminine ending in [ʁ] | *-ère(s)*/*-er(s)* | *boulangère* | *première* |
| Feminine ending in [z] | *-aise(s)*/*-ais* | *anglaise* | *français* |
|  | *-oise(s)*/*-ois* |  |  |
|  | *-euse(s)*/*-eux* | *amoureuse* | *heureuse* |
| Different f/m suffixes | *-esse(s)*/*-e(s)* |  |  |
|  | *-trice(s)*/*-teur(s)* | *dessinatrice*, *auteurs*, *provocateurs* | *négociatrice*, *réalisateurs*, *amateurs*, *interlocuteur* |
| Epicene words |  | *belge*, *libres*, *remarquables* | *suisse*, *disponibles* |

**B. Pronouns and determiners**

| Word type | F/m forms | Learning sentences | Test sentences |
| --- | --- | --- | --- |
| Personal pronoun | *elle(s)*/*il(s)* (subject) | *ils*, *il*, *elles* | *ils*, *il*, *elle* |
|  | *la(les)*/*le(s)* (direct object complement) | *le*, *la* | *le*, *la* |
|  | *lui(leur)* (indirect object complement, joint form) | *lui* | *lui* |
|  | *elle(s)*/*lui(eux)* (indirect object complement, disjunctive form) | *elle* | *elle* |
| Demonstrative pronoun | *celle(s)*/*celui(ceux)* | *celle* | *celui* |
| Possessive pronoun | *la(les) mienne(s)*/*le(s) mien(s)* | l*es miens* | *les siens* |
| Relative and interrogative pronouns | *laquelle(lesquelles)*/*lequel(lesquels)* |  |  |
| Definite article | *la(les)*/*le(s)* | *la*, *le* | *la*, *le* |
|  | *à la(aux)*/  *au(x)* |  |  |
|  | *de la(des)*/  *du(des)* |  |  |
| Indefinite article | *une(des)*/*un(des)* | *un*, *une* | *un*, *une* |
| Demonstrative determiner | *cette(s)*/*ce(s)* |  |  |
| Possessive determiner | *ma(mes)*/*mon(mes)* | *ta* | *ma* |
| Interrogative and exclamative determiner | *quelle(s)*/*quel(s)* | *quels* | *quels* |
| Indefinite determiner or pronoun | *aucune*/*aucun* | *aucun* | *aucun* |
|  | *chacune*/*chacun* |  |  |
|  | *quelqu’une(quelques unes)*/  *quelqu’un(quelques uns)* |  |  |
|  | *toute(s)*/*tout(tous)* | *tous* | *tous* |
|  | *certaine(s)*/*certain(s)* | *certains* | *certains* |
| Epicene words |  | *l’*, *les*, *des*, *ses* | *l’*, *les*, *des*, *s’*, *tes* |
