# Supplementary material for: Vowel alternation with final i offers an easy-to-learn morphological option for a sex-blind grammatical gender in French
Source: Front Psychol. 2024 Feb 22;15:1310475. doi: 10.3389/fpsyg.2024.1310475 (PMC10938390; doi:10.3389/fpsyg.2024.1310475)
Supplement: Supplementary file 1 [file Data_Sheet_1.ZIP › Data_Sheet/Data Sheet 3.docx]

***Supplementary Material***

**Supplementary Text 1**

# **Experimental design**

## **Introductory slides (original French text)**

Slide 1:

“Des extraterrestres parlent un langage qui n’indique jamais le sexe des personnes.

Pour des mots qui ont une forme différente au masculin et au féminin, par exemple :

il / elle, un / une, beau / belle,

musicien / musicienne,

ils ont inventé une troisième forme valable pour toutes les personnes, quel que soit leur sexe.”

Slide 2:

“Par contre ces extraterrestres utilisent les formes habituelles des mots lorsque ceux-ci ne marquent pas le sexe des personnes.

Par exemple : scénariste, libre, cosmonaute, les, votre…

Et les extraterrestres utilisent toujours les formes habituelles des mots quand ces mots se rapportent à des choses (qui n'ont pas de sexe…).

Par exemple : une belle bicyclette”

Slide 3:

“Les extraterrestres modifient donc le moins de mots possible : ils modifient uniquement les mots qui marquent le sexe des personnes.

Comme ces extraterrestres sont altruistes, vous essayez de maîtriser au mieux leur langage pour favoriser une alliance.

Pour vous exercer, 16 phrases sont d’abord proposées avec la traduction, puis une deuxième série de 16 sans la traduction.

Prenez votre temps : vous progressez à votre rythme…

À vous de jouer ! ;-)”

## Sentences to translate **(original French text)**

| n | Learning phase | Test phase |
| --- | --- | --- |
| 1 | la correspondante anglaise et un musicien belge | une représentante suisse et le mécanicien français |
| 2 | l’amoureuse comblée et un sportif ponctuel | l’invitée heureuse et un intellectuel actif |
| 3 | une nouvelle et meilleure dessinatrice | une nouvelle négociatrice bien supérieure |
| 4 | Quels auteurs provocateurs ! | Quels sont les réalisateurs amateurs ? |
| 5 | Ils ont tous été rassurés. | Ils furent tous admirés. |
| 6 | Ta belle cousine a mis une belle écharpe sur elle. | Ma belle voisine a acheté une belle voiture pour elle ! |
| 7 | Les humains naissent libres et égaux en droits. | Les écrivains se montrèrent disponibles et amicaux. |
| 8 | Des inconnues nous ont suivies dans cette grande ville. | Des amies sont venues prendre un déjeuner gourmand. |
| 9 | La mignonne boulangère chantait une chanson joyeuse. | La première championne parla d’un ton chaleureux. |
| 10 | Un tracteur américain tirait une lourde moissonneuse. | Sa volonté affirmée leur a valu de grands succès. |
| 11 | Je sais qu’il arrive car je le vois. | Il s’est trompé mais je le reprends. |
| 12 | Elles sont partisanes de la manière forte. | Elle parla à plusieurs paysannes. |
| 13 | Le voleur a été découvert. | Le joueur était un expert. |
| 14 | Il la regarda écrire et lui sourit. | Il lui serra la main en la regardant dans les yeux. |
| 15 | Certains de ses amis sont sensationnels mais les miens aussi sont remarquables. | Certains de tes collègues sont partis mais les siens sont tous restés. |
| 16 | Aucun ne voulut indiquer celle qui les avait guidés. | Celui qui était présent ne voulut écouter aucun interlocuteur. |

# **2 Generalized linear mixed models**

## 2.1 Models for all words

library(lme4)

f <- read.csv("Data Sheet 4.csv",skip = 3) ; dim(f) # 14320 11

f$sex <- as.factor(f$sex)

f$change <- as.factor(f$change)

f$solution <- as.factor(f$solution)

f$solution <- relevel(f$solution,ref="I")

f$POS <- as.factor(f$POS)

f$POS <- relevel(f$POS,ref="PART")

f$word <- as.factor(f$word)

f$id <- as.factor(f$id)

# Fixed effects = sex + age + solution + change

mm2 <- glmer(correct ~ sex + age + solution + change + (1|id) + (1|word),family = "binomial",control=glmerControl(optimizer = "bobyqa",optCtrl=list(maxfun=2e4)),data=f)

summary(mm2)

# Generalized linear mixed model fit by maximum likelihood (Laplace Approximation) [

# glmerMod]

# Family: binomial ( logit )

# Formula: correct ~ sex + age + solution + change + (1 | id) + (1 | word)

# Data: f

# Control: glmerControl(optimizer = "bobyqa", optCtrl = list(maxfun = 20000))

# AIC BIC logLik deviance df.resid

# 9765.4 9848.6 -4871.7 9743.4 14309

# Scaled residuals:

# Min 1Q Median 3Q Max

# -21.9688 0.0262 0.1350 0.3626 4.1552

# Random effects:

# Groups Name Variance Std.Dev.

# id (Intercept) 1.081 1.040

# word (Intercept) 1.186 1.089

# Number of obs: 14320, groups: id, 120; word, 120

# Fixed effects:

# Estimate Std. Error z value Pr(>|z|)

# (Intercept) 6.61191 0.42525 15.548 < 2e-16 ***

# sexMale -0.60457 0.20878 -2.896 0.00378 **

# age -0.01521 0.01026 -1.482 0.13822

# solutionA -1.67212 0.24532 -6.816 9.36e-12 ***

# solutionB -1.33803 0.24568 -5.446 5.15e-08 ***

# changeperson_epicene -2.28098 0.37260 -6.122 9.26e-10 ***

# changeperson_gendered -4.42679 0.27752 -15.951 < 2e-16 ***

# changething_epicene -0.61747 0.88927 -0.694 0.48746

# changething_gendered -1.70620 0.36146 -4.720 2.35e-06 ***

# ---

# Signif. codes: 0 ‘***’ 0.001 ‘**’ 0.01 ‘*’ 0.05 ‘.’ 0.1 ‘ ’ 1

# Correlation of Fixed Effects:

# (Intr) sexMal age soltnA soltnB chngprsn_p chngprsn_g chngthng_p

# sexMale -0.026

# age -0.686 -0.309

# solutionA -0.327 0.013 0.022

# solutionB -0.345 0.004 0.053 0.520

# chngprsn_pc -0.328 0.002 0.000 0.003 0.002

# chngprsn_gn -0.456 0.007 0.003 0.013 0.009 0.500

# chngthng_pc -0.137 0.000 0.000 0.001 0.000 0.154 0.209

# chngthng_gn -0.337 0.001 0.000 0.002 0.001 0.379 0.515 0.159

# The variable age has no significant effect on the response variable and is removed from the final model.

#---------------

# Fixed effects = sex + solution + change

mm3 <- glmer(correct ~ sex + solution + change + (1|id) + (1|word),family = "binomial",control=glmerControl(optimizer = "bobyqa",optCtrl=list(maxfun=2e4)),data=f)

summary(mm3)

# Generalized linear mixed model fit by maximum likelihood (Laplace Approximation) [

# glmerMod]

# Family: binomial ( logit )

# Formula: correct ~ sex + solution + change + (1 | id) + (1 | word)

# Data: f

# Control: glmerControl(optimizer = "bobyqa", optCtrl = list(maxfun = 20000))

# AIC BIC logLik deviance df.resid

# 9765.5 9841.2 -4872.8 9745.5 14310

# Scaled residuals:

# Min 1Q Median 3Q Max

# -22.0512 0.0263 0.1352 0.3623 4.1809

# Random effects:

# Groups Name Variance Std.Dev.

# id (Intercept) 1.103 1.050

# word (Intercept) 1.186 1.089

# Number of obs: 14320, groups: id, 120; word, 120

# Fixed effects:

# Estimate Std. Error z value Pr(>|z|)

# (Intercept) 6.1833 0.3105 19.911 < 2e-16 ***

# sexMale -0.7010 0.2004 -3.497 0.00047 ***

# solutionA -1.6669 0.2475 -6.735 1.64e-11 ***

# solutionB -1.3212 0.2476 -5.336 9.49e-08 ***

# changeperson_epicene -2.2810 0.3727 -6.120 9.37e-10 ***

# changeperson_gendered -4.4268 0.2776 -15.949 < 2e-16 ***

# changething_epicene -0.6176 0.8886 -0.695 0.48702

# changething_gendered -1.7062 0.3614 -4.721 2.35e-06 ***

# ---

# Signif. codes: 0 ‘***’ 0.001 ‘**’ 0.01 ‘*’ 0.05 ‘.’ 0.1 ‘ ’ 1

# Correlation of Fixed Effects:

# (Intr) sexMal soltnA soltnB chngprsn_p chngprsn_g chngthng_p

# sexMale -0.345

# solutionA -0.431 0.020

# solutionB -0.427 0.021 0.520

# chngprsn_pc -0.449 0.002 0.003 0.002

# chngprsn_gn -0.623 0.008 0.013 0.009 0.500

# chngthng_pc -0.188 0.000 0.001 0.001 0.155 0.210

# chngthng_gn -0.462 0.001 0.002 0.002 0.379 0.515 0.159

## **2.2 Models for POS categories with gender agreement**

### 2.2.1 Model for determiners

ff <- subset(f,change != "no_agreement") ; dim(ff) # 10142 11

ff$change <- relevel(ff$change,ref="person_gendered")

det <- subset(ff,POS == "DET") ; dim(det) # 2747 11

mdet <- glmer(correct ~ sex + age + solution + change + (1|id) + (1|word),family = "binomial",control=glmerControl(optimizer = "bobyqa",optCtrl=list(maxfun=2e4)),data=det)

summary(mdet)

# Generalized linear mixed model fit by maximum likelihood (Laplace Approximation) [

# glmerMod]

# Family: binomial ( logit )

# Formula: correct ~ sex + age + solution + change + (1 | id) + (1 | word)

# Data: det

# Control: glmerControl(optimizer = "bobyqa", optCtrl = list(maxfun = 20000))

# AIC BIC logLik deviance df.resid

# 2326.5 2385.7 -1153.2 2306.5 2737

# Scaled residuals:

# Min 1Q Median 3Q Max

# -8.7620 0.1244 0.2794 0.4626 2.1193

# Random effects:

# Groups Name Variance Std.Dev.

# id (Intercept) 0.7639 0.8740

# word (Intercept) 0.6013 0.7754

# Number of obs: 2747, groups: id, 120; word, 23

# Fixed effects:

# Estimate Std. Error z value Pr(>|z|)

# (Intercept) 2.587614 0.442491 5.848 4.98e-09 ***

# sexMale -0.533252 0.206558 -2.582 0.009834 **

# age -0.020954 0.009933 -2.110 0.034900 *

# solutionA -0.832646 0.244474 -3.406 0.000660 ***

# solutionB -0.825306 0.244899 -3.370 0.000752 ***

# changeperson_epicene 0.886925 0.412876 2.148 0.031701 *

# changething_epicene 2.925022 0.721569 4.054 5.04e-05 ***

# changething_gendered 1.188682 0.458374 2.593 0.009507 **

# ---

# Signif. codes: 0 ‘***’ 0.001 ‘**’ 0.01 ‘*’ 0.05 ‘.’ 0.1 ‘ ’ 1

# Correlation of Fixed Effects:

# (Intr) sexMal age soltnA soltnB chngp_ chngthng_p

# sexMale -0.037

# age -0.646 -0.306

# solutionA -0.324 0.017 0.030

# solutionB -0.345 0.018 0.058 0.540

# chngprsn_pc -0.389 -0.005 -0.004 -0.007 -0.007

# chngthng_pc -0.217 -0.007 -0.005 -0.009 -0.009 0.246

# chngthng_gn -0.348 -0.006 -0.005 -0.009 -0.009 0.385 0.222

### 2.2.2 Model for **nouns**

nom <- subset(ff,POS == "NOUN") ; dim(nom) # 2631 11

mnom <- glmer(correct ~ sex + age + solution + change + (1|id) + (1|word),family = "binomial",control=glmerControl(optimizer = "bobyqa",optCtrl=list(maxfun=2e4)),data=nom)

summary(mnom)

# Generalized linear mixed model fit by maximum likelihood (Laplace Approximation) [

# glmerMod]

# Family: binomial ( logit )

# Formula: correct ~ sex + age + solution + change + (1 | id) + (1 | word)

# Data: nom

# Control: glmerControl(optimizer = "bobyqa", optCtrl = list(maxfun = 20000))

# AIC BIC logLik deviance df.resid

# 2255.3 2308.2 -1118.6 2237.3 2622

# Scaled residuals:

# Min 1Q Median 3Q Max

# -13.5569 -0.4891 0.1289 0.4373 4.3670

# Random effects:

# Groups Name Variance Std.Dev.

# id (Intercept) 1.1802 1.0864

# word (Intercept) 0.6181 0.7862

# Number of obs: 2631, groups: id, 120; word, 22

# Fixed effects:

# Estimate Std. Error z value Pr(>|z|)

# (Intercept) 1.77644 0.46381 3.830 0.000128 ***

# sexMale -0.45844 0.24047 -1.906 0.056596 .

# age -0.01027 0.01185 -0.866 0.386287

# solutionA -2.02595 0.28646 -7.072 1.52e-12 ***

# solutionB -1.82206 0.28623 -6.366 1.94e-10 ***

# changeperson_epicene 1.39312 0.84868 1.642 0.100689

# changething_gendered 4.27587 0.42969 9.951 < 2e-16 ***

# ---

# Signif. codes: 0 ‘***’ 0.001 ‘**’ 0.01 ‘*’ 0.05 ‘.’ 0.1 ‘ ’ 1

# Correlation of Fixed Effects:

# (Intr) sexMal age soltnA soltnB chngp_

# sexMale -0.028

# age -0.724 -0.308

# solutionA -0.351 0.026 0.024

# solutionB -0.373 0.017 0.055 0.539

# chngprsn_pc -0.114 -0.003 -0.001 -0.012 -0.011

# chngthng_gn -0.211 -0.016 -0.010 -0.046 -0.041 0.137

### 2.2.3 Model for **adjectives**

adj <- subset(ff,POS == "ADJ") ; dim(adj) # 2858 11

madj <- glmer(correct ~ sex + age + solution + change + (1|id) + (1|word),family = "binomial",control=glmerControl(optimizer = "bobyqa",optCtrl=list(maxfun=2e4)),data=adj)

summary(madj)

# Generalized linear mixed model fit by maximum likelihood (Laplace Approximation) [

# glmerMod]

# Family: binomial ( logit )

# Formula: correct ~ sex + age + solution + change + (1 | id) + (1 | word)

# Data: adj

# Control: glmerControl(optimizer = "bobyqa", optCtrl = list(maxfun = 20000))

# AIC BIC logLik deviance df.resid

# 2857.7 2911.4 -1419.9 2839.7 2849

# Scaled residuals:

# Min 1Q Median 3Q Max

# -10.2456 -0.5739 0.1852 0.5433 4.1241

# Random effects:

# Groups Name Variance Std.Dev.

# id (Intercept) 1.367 1.1690

# word (Intercept) 0.504 0.7099

# Number of obs: 2858, groups: id, 120; word, 24

# Fixed effects:

# Estimate Std. Error z value Pr(>|z|)

# (Intercept) 1.97063 0.46072 4.277 1.89e-05 ***

# sexMale -0.71483 0.24928 -2.868 0.00414 **

# age -0.01761 0.01228 -1.434 0.15168

# solutionA -1.90786 0.29516 -6.464 1.02e-10 ***

# solutionB -1.52664 0.29501 -5.175 2.28e-07 ***

# changeperson_epicene 2.51700 0.57115 4.407 1.05e-05 ***

# changething_gendered 2.71915 0.39644 6.859 6.93e-12 ***

# ---

# Signif. codes: 0 ‘***’ 0.001 ‘**’ 0.01 ‘*’ 0.05 ‘.’ 0.1 ‘ ’ 1

# Correlation of Fixed Effects:

# (Intr) sexMal age soltnA soltnB chngp_

# sexMale -0.029

# age -0.754 -0.308

# solutionA -0.363 0.026 0.023

# solutionB -0.384 0.017 0.052 0.533

# chngprsn_pc -0.109 -0.009 -0.006 -0.018 -0.014

# chngthng_gn -0.156 -0.015 -0.010 -0.027 -0.021 0.153

### 2.2.4 Model for **pronouns**

pron <- subset(ff,POS == "PRON") ; dim(pron) # 1906 11

mpron <- glmer(correct ~ sex + age + solution + change + (1|id) + (1|word),family = "binomial",control=glmerControl(optimizer = "bobyqa",optCtrl=list(maxfun=2e4)),data=pron)

summary(mpron)

# Generalized linear mixed model fit by maximum likelihood (Laplace Approximation) [

# glmerMod]

# Family: binomial ( logit )

# Formula: correct ~ sex + age + solution + change + (1 | id) + (1 | word)

# Data: pron

# Control: glmerControl(optimizer = "bobyqa", optCtrl = list(maxfun = 20000))

# AIC BIC logLik deviance df.resid

# 1658.2 1702.6 -821.1 1642.2 1898

# Scaled residuals:

# Min 1Q Median 3Q Max

# -6.9429 -0.3306 0.2233 0.4829 3.2805

# Random effects:

# Groups Name Variance Std.Dev.

# id (Intercept) 0.8734 0.9346

# word (Intercept) 1.7039 1.3053

# Number of obs: 1906, groups: id, 120; word, 16

# Fixed effects:

# Estimate Std. Error z value Pr(>|z|)

# (Intercept) 2.66606 0.55970 4.763 1.90e-06 ***

# sexMale -0.52563 0.22752 -2.310 0.020872 *

# age -0.01788 0.01107 -1.615 0.106226

# solutionA -1.68209 0.27230 -6.177 6.52e-10 ***

# solutionB -0.88691 0.27294 -3.250 0.001156 **

# changeperson_epicene 2.72311 0.75950 3.585 0.000337 ***

# ---

# Signif. codes: 0 ‘***’ 0.001 ‘**’ 0.01 ‘*’ 0.05 ‘.’ 0.1 ‘ ’ 1

# Correlation of Fixed Effects:

# (Intr) sexMal age soltnA soltnB

# sexMale -0.033

# age -0.569 -0.306

# solutionA -0.303 0.033 0.038

# solutionB -0.310 0.020 0.060 0.552

# chngprsn_pc -0.360 -0.009 -0.009 -0.025 -0.011

## **2.3 Models for all words with gender agreement**

# Fixed effects = sex + age + solution + change + POS

ff <- subset(f,change != "no_agreement") ; dim(ff) # 10142 11

ff$change <- relevel(ff$change,ref="thing_epicene")

ff$POS <- relevel(ff$POS,ref="NOUN")

mm4 <- glmer(correct ~ sex + age + solution + change + POS + (1|id) + (1|word),family = "binomial",control=glmerControl(optimizer = "bobyqa",optCtrl=list(maxfun=2e4)),data=ff)

summary(mm4)

# Generalized linear mixed model fit by maximum likelihood (Laplace Approximation) ['glmerMod']

# Family: binomial ( logit )

# Formula: correct ~ sex + age + solution + change + POS + (1 | id) + (1 | word)

# Data: ff

# Control: glmerControl(optimizer = "bobyqa", optCtrl = list(maxfun = 20000))

# AIC BIC logLik deviance df.resid

# 8867.1 8961.1 -4420.6 8841.1 10129

# Scaled residuals:

# Min 1Q Median 3Q Max

# -10.5323 -0.4824 0.1980 0.4891 4.2205

# Random effects:

# Groups Name Variance Std.Dev.

# id (Intercept) 1.082 1.040

# word (Intercept) 0.937 0.968

# Number of obs: 10142, groups: id, 120; word, 85

# Fixed effects:

# Estimate Std. Error z value Pr(>|z|)

# (Intercept) 5.85294 0.91928 6.367 1.93e-10 ***

# sexMale -0.58599 0.20934 -2.799 0.00512 **

# age -0.01673 0.01030 -1.624 0.10435

# solutionA -1.75641 0.24612 -7.136 9.57e-13 ***

# solutionB -1.42485 0.24640 -5.783 7.35e-09 ***

# changeperson_epicene -1.78549 0.84008 -2.125 0.03356 *

# changeperson_gendered -3.69038 0.82528 -4.472 7.76e-06 ***

# changething_gendered -0.82294 0.84553 -0.973 0.33041

# POSADJ -0.36905 0.29940 -1.233 0.21771

# POSDET 0.25318 0.32138 0.788 0.43081

# POSPRON 0.91980 0.34974 2.630 0.00854 **

# ---

# Signif. codes: 0 ‘***’ 0.001 ‘**’ 0.01 ‘*’ 0.05 ‘.’ 0.1 ‘ ’ 1

# Correlation of Fixed Effects:

# (Intr) sexMal age soltnA soltnB chngprsn_p chngprsn_g chngt_ POSADJ POSDET

# sexMale -0.011

# age -0.318 -0.308

# solutionA -0.151 0.012 0.022

# solutionB -0.159 0.003 0.054 0.521

# chngprsn_pc -0.833 0.001 0.000 0.000 0.000

# chngprsn_gn -0.886 0.002 0.000 0.003 0.002 0.927

# chngthng_gn -0.861 0.000 -0.001 -0.001 -0.001 0.894 0.936

# POSADJ -0.175 0.001 0.000 0.001 0.000 -0.012 -0.003 0.021

# POSDET -0.346 -0.001 -0.001 -0.003 -0.003 0.109 0.215 0.204 0.501

# POSPRON -0.162 -0.002 -0.001 -0.005 -0.004 -0.057 0.002 0.072 0.473 0.472

# The variable age has no significant effect on the response variable and is eliminated from the final model.

#---------------

# Fixed effects = sex + solution + change + POS

mm5 <- glmer(correct ~ sex + solution + change + POS + (1|id) + (1|word),family = "binomial",control=glmerControl(optimizer = "bobyqa",optCtrl=list(maxfun=2e4)),data=ff)

summary(mm5)

# Generalized linear mixed model fit by maximum likelihood (Laplace Approximation) ['glmerMod']

# Family: binomial ( logit )

# Formula: correct ~ sex + solution + change + POS + (1 | id) + (1 | word)

# Data: ff

# Control: glmerControl(optimizer = "bobyqa", optCtrl = list(maxfun = 20000))

# AIC BIC logLik deviance df.resid

# 8867.8 8954.4 -4421.9 8843.8 10130

# Scaled residuals:

# Min 1Q Median 3Q Max

# -10.4419 -0.4839 0.1981 0.4889 4.2521

# Random effects:

# Groups Name Variance Std.Dev.

# id (Intercept) 1.109 1.053

# word (Intercept) 0.937 0.968

# Number of obs: 10142, groups: id, 120; word, 85

# Fixed effects:

# Estimate Std. Error z value Pr(>|z|)

# (Intercept) 5.3819 0.8725 6.168 6.92e-10 ***

# sexMale -0.6920 0.2014 -3.436 0.000591 ***

# solutionA -1.7508 0.2488 -7.036 1.97e-12 ***

# solutionB -1.4064 0.2488 -5.653 1.58e-08 ***

# changeperson_epicene -1.7856 0.8412 -2.123 0.033782 *

# changeperson_gendered -3.6904 0.8261 -4.467 7.93e-06 ***

# changething_gendered -0.8231 0.8464 -0.973 0.330800

# POSADJ -0.3690 0.2994 -1.232 0.217802

# POSDET 0.2531 0.3213 0.788 0.430821

# POSPRON 0.9197 0.3498 2.629 0.008561 **

# ---

# Signif. codes: 0 ‘***’ 0.001 ‘**’ 0.01 ‘*’ 0.05 ‘.’ 0.1 ‘ ’ 1

# Correlation of Fixed Effects:

# (Intr) sexMal soltnA soltnB chngprsn_p chngprsn_g chngt_ POSADJ POSDET

# sexMale -0.122

# solutionA -0.152 0.020

# solutionB -0.151 0.020 0.520

# chngprsn_pc -0.879 0.000 0.000 -0.001

# chngprsn_gn -0.934 0.002 0.002 0.001 0.928

# chngthng_gn -0.908 -0.001 -0.002 -0.002 0.894 0.936

# POSADJ -0.184 0.001 0.001 0.000 -0.012 -0.004 0.021

# POSDET -0.365 -0.002 -0.003 -0.003 0.109 0.214 0.203 0.501

# POSPRON -0.171 -0.002 -0.005 -0.004 -0.057 0.001 0.071 0.473 0.472

**Supplementary Figure 1**


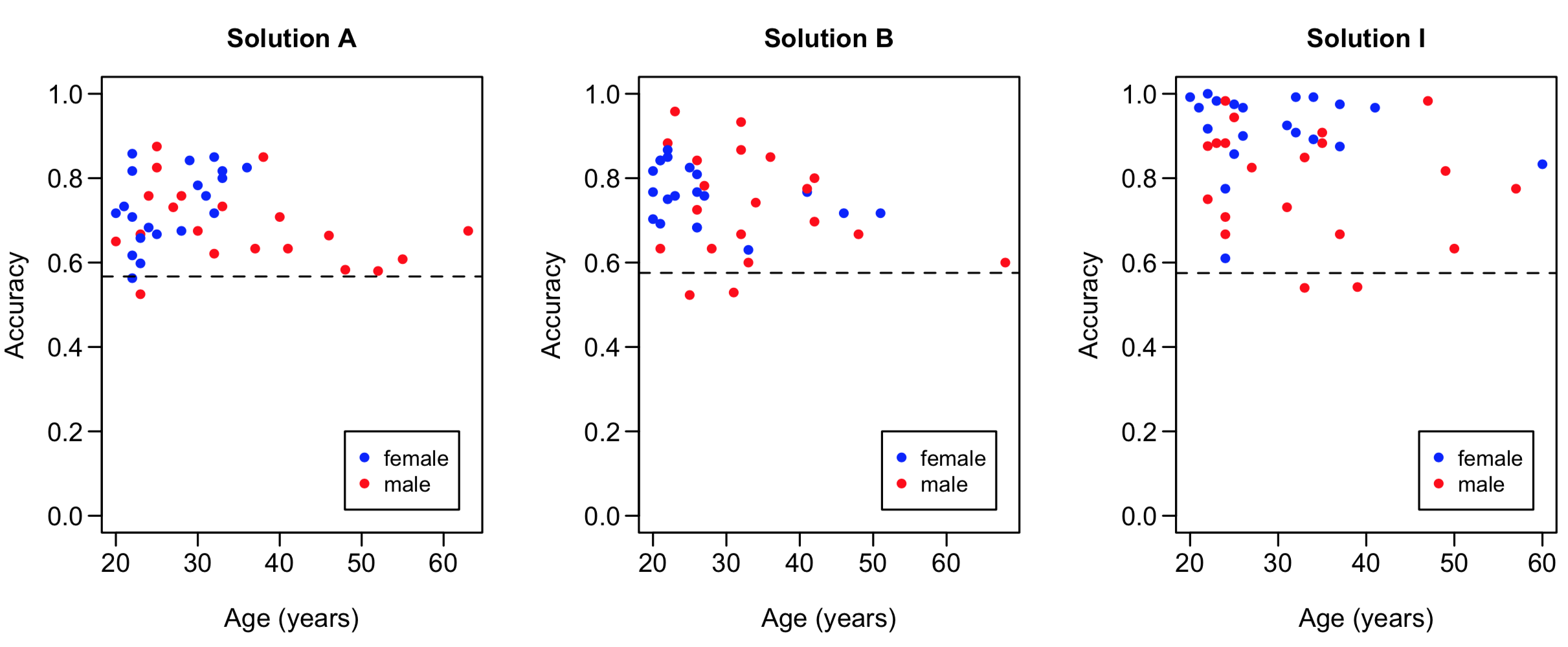
**Supplementary Figure 1.** Accuracy of the participants’ answers as a function of their age. The dashed lines indicate the baseline accuracy scores as defined in the main text.
